# Digitally Assisted Mindfulness in Training Self-Regulation Skills for Sustainable Mental Health: A Systematic Review

**DOI:** 10.3390/bs13121008

**Published:** 2023-12-10

**Authors:** Eleni Mitsea, Athanasios Drigas, Charalabos Skianis

**Affiliations:** 1 Net Media Lab & Mind & Brain R&D, Institute of Informatics & Telecommunications, National Centre of Scientific Research ‘Demokritos’ Athens, Agia Paraskevi, 15341 Athens, Greece; dr@iit.demokritos.gr; 2 Department of Information and Communication Systems Engineering, University of Aegean, 82300 Mytilene, Greece; cskianis@aegean.gr

**Keywords:** digital mindfulness, sustainable wellness, self-regulation, virtual reality, augmented reality, mixed reality, extended reality, metaverse, artificial intelligence, internet of things, chatbots, chatGPT, brain-sensing headbands, biofeedback

## Abstract

The onset of the COVID-19 pandemic has led to an increased demand for mental health interventions, with a special focus on digitally assisted ones. Self-regulation describes a set of meta-skills that enable one to take control over his/her mental health and it is recognized as a vital indicator of well-being. Mindfulness training is a promising training strategy for promoting self-regulation, behavioral change, and mental well-being. A growing body of research outlines that smart technologies are ready to revolutionize the way mental health training programs take place. Artificial intelligence (AI); extended reality (XR) including virtual reality (VR), augmented reality (AR), and mixed reality (MR); as well as the advancements in brain computer interfaces (BCIs) are ready to transform these mental health training programs. Mindfulness-based interventions assisted by smart technologies for mental, emotional, and behavioral regulation seem to be a crucial yet under-investigated issue. The current systematic review paper aims to explore whether and how smart technologies can assist mindfulness training for the development of self-regulation skills among people at risk of mental health issues as well as populations with various clinical characteristics. The PRISMA 2020 methodology was utilized to respond to the objectives and research questions using a total of sixty-six experimental studies that met the inclusion criteria. The results showed that digitally assisted mindfulness interventions supported by smart technologies, including AI-based applications, chatbots, virtual coaches, immersive technologies, and brain-sensing headbands, can effectively assist trainees in developing a wide range of cognitive, emotional, and behavioral self-regulation skills, leading to a greater satisfaction of their psychological needs, and thus mental wellness. These results may provide positive feedback for developing smarter and more inclusive training environments, with a special focus on people with special training needs or disabilities.

## 1. Introduction

The onset of the COVID-19 pandemic has led to an increased demand for mental health training programs. At the same time, a significant percentage of mental health practitioners acknowledge telehealth services and digitally assisted interventions as a key component of 21st-century healthcare [1,2]. Digital technologies are considered promising assistive tools not only for trainers but also for trainees to be actively engaged in mental health training programs and learn how to effectively apply self-management strategies for strengthening mental and emotional well-being [3]. Digital mental health tools are gaining an increasing focus because of their potential to bridge barriers by providing access to high-quality mental health training programs and individualized interventions among people with special training needs such as people with disabilities [4].

Self-regulation refers to a set of meta-skills that allows one to be aware, monitor, and adaptively control his/her cognitive functions, thoughts, emotions, and behaviors to achieve psychophysiological balance. Self-regulated individuals can evaluate whether their actions are consistent with internal and external demands, being able to redirect themselves whenever they observe discrepancies [5]. Self-regulation capacity is considered an indicator of mental health and psychological wellbeing. In addition, the research provides evidence that difficulties in self-regulation (i.e., impulsivity, rumination) predict various clinical conditions in mental health [6,7]. Thus, self-regulation is recognized as a central avenue through which mindfulness may boost mental health and well-being [8].

Mindfulness-based interventions integrate a wide range of mental health training techniques grounded in the core principles of mindfulness theory. Designed to voluntarily direct one’s attention to the present moment in a way that is open, discerning, and non-judgmental, mindfulness-based interventions primarily aim to induce a mental state of active awareness employing mindful attention as the main driver to achieve effortless self-regulation [9]. A growing body of research indicates that mindfulness-based interventions can be a promising avenue for training self-regulation skills [10,11,12]. By strengthening the sense of active awareness along with the meta-ability to monitor and regulate, moment by moment, the current state of mind, mindfulness intends to achieve a physiological balance and harmonize the cognitive and behavioral reactions to unexpected or stressful internal or external events. However, such training requires commitment and long-term practice [13].

Mindfulness-based interventions have already revealed promising outcomes for improving mental health [14]. Mindfulness techniques have been successfully integrated into well-established psychotherapeutic methods [15,16], providing promising outcomes for people with self-regulation deficits including those with neurodevelopmental disorders, anxiety disorders, and emotional and behavioral disorders [17,18,19]. Thus, mindfulness interventions seem to be a promising tool for developing self-management skills and strengthening psychological balance.

Information and Communication Technologies (ICTs) are already effectively utilized as assistive tools in various training interventions for mental and emotional well-being [20]. A growing number of studies reason that innovative digital technologies may offer potential advantages over conventional training programs in terms of accessibility, standardization, personalization, and efficacy [21]. Such advantages may be crucial especially for people with special training needs such as people with mental health disorders [22]. In that vein, digital aids and interactive technologies are of particular importance for the widespread dissemination of mindfulness training [21,23].

Smart technologies refer to a set of networked devices, systems, and applications that make use of AI, the Internet of Things (IoT), and other advanced technologies that interact, enabling automatic and adaptive functioning as well as remote accessibility or operation from any location [24]. A growing body of research outlines that smart technologies are ready to reshape the way mental health training programs take place [25]. One of the significant advantages that smart technologies will provide is that people will have unlimited access to high-quality training programs adapted to their personal needs and preferences. AI already has a crucial role in digital mental health programs [26,27]. AI applications are expected to radically change mental health interventions, especially those addressed to people with special training needs [28]. XR including VR, AR, and MR technologies are gradually gaining ground in mental health interventions [25,29]. A growing body of experimental studies explores the role of immersive technologies in innovating intervention programs that aim to induce relaxation and develop self-regulation skills among people with mental health conditions [30]. The growing interest in neurocognitive enhancement has also directed the research focus on neurofeedback technologies with a special focus on non-invasive smart brain-sensing headbands [31].

Although everyone has the potential to practice mindfulness, mindfulness training interventions can be highly effortful and challenging, especially for novices [32]. Trainees often find it difficult to keep their attention active and monitor their progress, which results in decreased motivation and the early departure from intervention programs [33]. In addition, it is quite difficult for trainees to recognize what state of mind they are in [34]. For children, practicing mindfulness may be quite challenging because there are no obvious indicators of awareness that trainers or therapists may use to provide them feedback [35]. Face-to-face interventions are often quite challenging due to various geographical and financial constraints. In addition, novice trainees find it more difficult to maintain a disciplined and/or systematic practice, especially in real-life situations. Thus, they tend to give up practice [32,36]. Digitally assisted mindfulness programs may help individuals develop their meditation practice more rapidly and effectively [32].

Although there is a growing interest in creating digital-assisted mindfulness interventions for promoting mental health and psychological balance, the research is still in its early stages. The research regarding the new role of smart technologies in such mental health training programs remains quite limited [32]. In addition, fewer studies examine the impact of digitally assisted mindfulness practices on skills training, especially those that ensure mental and emotional regulation and well-being. These skills include a wide range of meta-cognitive and meta-emotional skills such as attentional control, emotional control, and inhibition control [37].

In this study, we hypothesize that smart technologies could actively contribute to minimizing the challenges that people, especially those with mental health issues, face in conventional mindfulness practices. In addition, we expect that smart technologies with their special features can take the training process a step forward, providing both trainers and trainees with more opportunities to develop the meta-cognitive and emotional intelligence skills needed to be self-regulated, autonomous, and self-satisfied.

Specifically, this systematic review aims to answer the following questions:

1. Can smart technologies effectively assist mindfulness programs for both healthy populations and clinical populations?
2. What types of smart technologies are being used in mindfulness training?
3. Can digitally assisted mindfulness interventions effectively support trainees in developing the self-regulation meta-skills needed for mental and emotional well-being?

According to our knowledge, this review paper is one of the few that focuses on the effectiveness of emerging technologies in assisting mindfulness training in both populations with clinical and non-clinical symptoms. In addition, it co-examines the role of smart technologies in assisting the training of self-regulation skills needed for sustainable mental and psychological health. This paper intends to contribute to the discussion with regard to the creation of smart, inclusive, and sustainable training environments for promoting mental health and psychological balance.

## 2. Materials and Methods

### 2.1. Study Design

This study complied with the guidelines of the Preferred Reporting Items for Systematic Reviews and Meta-Analyses (PRISMA) statement. The PRISMA statement guides the researchers to identify, select, evaluate, and synthesize the studies included in a systematic review. The PRISMA 2020 checklist (available in the Supplementary Material was employed to assure that the required methodological steps were followed [38]. The protocol for the systematic review was registered with Open Science Framework (osf.io/y9ctd) [39]. From March 2023 to August 2023, a group of three researchers carried out a systematic research effort.

### 2.2. Eligibility Criteria

In our search, we mainly focused on experimental studies with a special focus on randomized controlled trials. However, we also included quasi-experimental studies provided that they fit the scope of the current review as well as the quality criteria required for being included. Systematic reviews, meta-analyses, and book chapters were excluded. Protocols and design frameworks without testing the feasibility of the proposed intervention were also excluded.

With regard to the type of population, healthy people who are at risk of developing mental health issues (i.e., due to factors such as living and working in stressful and demanding environments) were included. As an example, we can mention workers, older people, and students. In addition, in this study, populations with a diagnosis of mental health problems were included. Special emphasis was given to people who experience difficulties in self-regulation (i.e., neurodevelopmental disorders, behavioral and mood disorders). With regard to the type of intervention, the current review exclusively focused on mindfulness interventions. A requirement was the employment of assistive technologies, and especially smart technologies including AI, XR, VR, AR, biofeedback technologies, and BCIs. Among the inclusion criteria was the investigation of self-regulation skills. Thus, we selected studies that trained and evaluated the effectiveness of digitally assisted mindfulness interventions in the development of skills that allow one to develop self-regulated behaviors such as the ability to control attention, manage the intensity of emotions, and effectively inhibit impulses. In Table 1, the inclusion and exclusion criteria are listed.

### 2.3. Information Sources

We conducted our research with the use of the following four academic search engines: Web of Science, Scopus, Pubmed, and Google Scholar. These databases are trustworthy and among the largest academic databases with peer-reviewed and high-quality studies. They are widely accepted as valuable tools for conducting systematic reviews. They are easy-to-use interfaces that allow researchers to have quick access to relevant scientific papers. These academic databases offer a wide range of search tools accelerating the process of paper identification. In addition, these databases fit with the scope of this paper as well as the research questions, because they incorporate studies in disciplines of cognitive science, cognitive psychology, computer sciences, and digital health.

### 2.4. Search Strategy

This research was limited to papers published after 2010 until August 2023. The main search terms used in our search strategy included terms related to mindfulness training such as mindfulness breathing, guided meditation, positive visualizations, focused attention, open monitoring, guided imagery, and body scanning. In addition, the search also included keywords related to smart technologies including artificial intelligence, AI conversational agents, ChatGPT, mixed reality, augmented reality, virtual reality, virtual companions, virtual coaches, the metaverse, and biofeedback and neurofeedback technologies. Moreover, we narrowed down our research searching for terms related to self-regulation skills such as attentional control, emotional control, impulse control, self-observation, stress management, and adaptability. The chosen databases allowed the employment of boolean operators that played the role of conjunctions to integrate the central searching keywords. Moreover, the database search limit allowed us to further narrow the results to retrieve the most relevant studies to our research question. Table 2 illustrates the general search strategy followed because each database has some differences with regard to the advanced searching processes.

### 2.5. Selection Processes

Initially, we determined the eligibility criteria according to which we made our search across the four academic databases. By using the search tools and the filters provided by the databases, we collected the candidate studies for further processing. We prioritized studies that included the predefined keywords in the title and the abstract. At the abstract/title screening stage, after removing deduplicated studies, the references that were not under the eligibility criteria were excluded. The studies that met the inclusion criteria were kept for full-text screening. After the retrieval of the full-text documents, we made an in-depth assessment of the content of the papers. The remaining papers were examined in detail at the full-text screening stage. At this stage of screening, we took into consideration parameters such as the methodology used in this study. Two reviewers independently selected eligible papers, deciding whether they accepted, rejected, or were unsure about the inclusion of the study, providing relevant reasons. If screeners were unsure or disagreed, then a final decision was made after discussion and the support of the third reviewer. After the selection and the valuation of the studies, we tried to synthesize the results. A qualitative synthesis was conducted, summarizing, analyzing, and assessing the body of evidence included in this review.

### 2.6. Data Collection

In the next stage of processing, data were gathered from each chosen paper by two reviewers independently. We further processed each study to collect the data needed including the author’s information, the sample’s characteristics (number of participants, gender, and mean age), the type of mindfulness training, the design of the research, the type of assistive technology, the period of intervention, the measurements, and the main findings. The data extracted from each paper were organized to facilitate the interpretation processes (Table A1, Table A2 and Table A3 in Appendix A).

### 2.7. Study Risk of Bias Assessment

To evaluate the risk of bias in the randomized controlled trials, the Cochrane Collaboration’s Risk of Bias Version 2 tool (ROB-2) was employed. RoB-2 describes a set of five bias domains. Specifically, it examines bias arising from the randomization process. In addition, it seeks deviations from the planned intervention and bias due to missing outcome data. ROB-2 also assesses bias in the measurement of the outcome, as well as bias due to the selection of the reported results. The evaluators could choose among the following three risk-of-bias judgments: low risk of bias, some concerns, and high risk of bias [40].

The remaining non-randomized studies were assessed with the Risk of Bias for Non-randomized Studies of Interventions tool (ROBINS-I) that introduces the following seven domains of possible bias: confounding, selection of sample, intervention, deviations from planned interventions, missing data, measurement of outcomes, and selection of the reported results. The classification for risk-of-bias judgments was a “low risk”, “moderate risk”, “serious risk”, and “critical risk” of bias [41].

Each paper was independently evaluated by two authors, and conflicts were resolved with the support of the third reviewer.

## 3. Background Knowledge

### 3.1. Laying the Building Blocks for Self-Regulation in Mindfulness Training

#### The Special Role of Metacognition, Emotional Intelligence, and Motivation Theory

New evidence has shown that mindfulness improves higher-order neurocognitive control operations inseparably associated with self-regulation. Many researchers have concluded that mindfulness training is considered a potential avenue for developing a wide range of self-regulation skills [42].

More specifically, a fundamental component of mindfulness training is considered the enhancement in attentional self-regulation skills [43]. Practitioners are systematically trained to voluntarily monitor, control, and adapt their attention, starting with paying non-judgmental attention to the present moment experience. By learning strategies to monitor and regulate attentional operations, trainees gradually develop other forms of regulation including the ability to self-regulate maladaptive behaviors [44,45].

Mindfulness training improves self-observation skills, the active monitoring of one’s own inner “world” (i.e., intentions, expectations, emotions, beliefs, and behaviors) [46]. Self-observation allows one to be aware and reflect on his/her thoughts and feelings as well as to be in contact with their surroundings including the social environment [47].

Mindfulness encourages practitioners to remain in a state of non-automatic reaction, boosting another fundamental self-regulation skill, namely inhibition control [48]. Inhibition control allows trainees to control their impulses or any other automatic or dominating behavioral reaction to external or internal stimuli [49].

Research has indicated that mindfulness training can help trainees to develop stress management skills. According to Kadziolka et al. [50], systematic practice in mindfulness improves trainees’ ability to engage in self-regulatory physiological responses, which is closely associated with the efficacious downregulation of stress. At the same time, practitioners improve their ability to avoid costly physiological activations in response to challenging emotional situations.

Mindfulness encourages the development of emotional intelligence skills that are responsible for being self-controlled [51]. Specifically, the use of mindfulness techniques enables trainees to perceive the emotional signals and manage them effectively with an attitude of acceptance, without trying to change them [52]. Mindfulness training helps practitioners to perceive emotional experiences with clarity while encouraging them to focus on the positive rather than negative aspects of these experiences [51,53].

Mindfulness training has the potential to raise trainees’ intrinsic motivation, thus leading them to more autonomous forms of self-regulation [54]. More specifically, mindfulness training requires the trainee to deal with true desires, authentic interests, and values, as well as pursue internal goals. This process supports the fulfillment of fundamental psychological needs, fosters the internalization of positive thoughts and behaviors, and leads trainees to more awakened, self-determined, and autonomous forms of self-regulation, thus increasing the possibility of experiencing overall wellness [55].

**Figure 1:**
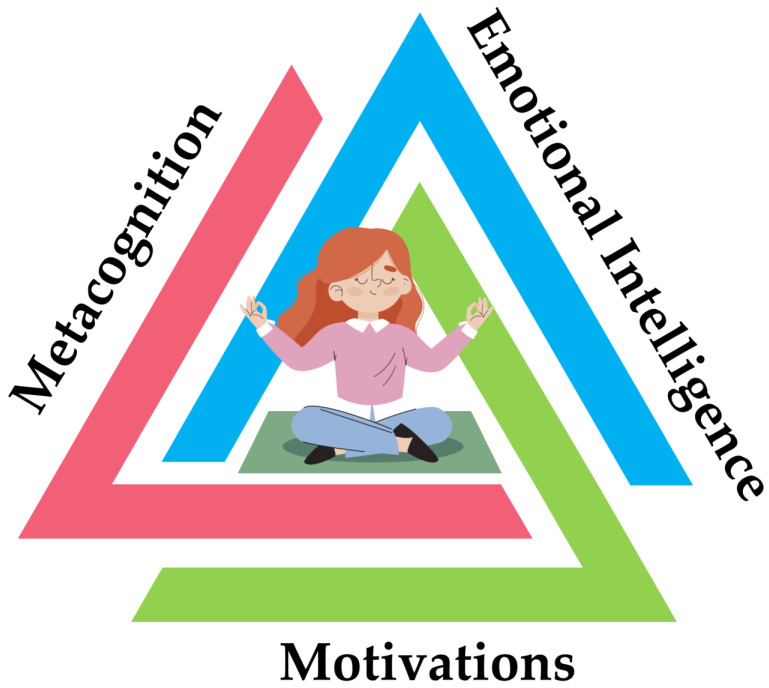
Metacognition, emotional intelligence, and motivations constitute the building blocks for self-regulation in mindfulness training.

**Figure 2:**
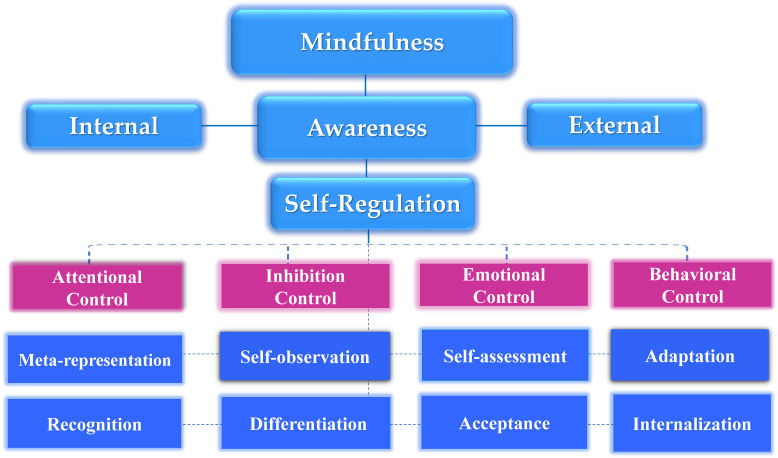
The main metacognitive components of mindfulness training.

Thus, it is obvious that mindfulness is increasingly viewed as a training strategy that activates a set of self-regulation skills closely associated with the theories of metacognition, emotional intelligence, and motivation (Figure 1 and Figure 2) [37,51,55]. However, the efficacy of digitally assisted mindfulness in the domains of self-regulation, especially among people with clinical conditions, remains to a significant extent underexplored. Figure 1 signifies that theories of metacognition, emotional intelligence, and motivation theory constitute core and complementary components in mindfulness training. Figure 2 presents the core regulatory meta-competences involved in mindfulness training.

### 3.2. Smart Technologies: The New Eve in Mental Health Interventions

#### 3.2.1. The Potential Benefits of Artificial Intelligence in Mindfulness Training

AI is expected to play a key role in future mental health programs by providing innovative tools and methods for designing more accessible and personalized coaching [28,56]. AI through machine learning algorithms can offer personalized suggestions, record progress, and provide users with real-time feedback. Identifying cognitive and behavioral patterns, AI-powered apps can adjust according to the users’ real-time data. AI can recognize users’ personal preferences and interests and provide appropriate triggers to boost users’ motivation to continue efforts toward training goals. AI mental health applications can help patients who avoid seeking health-related advice because of stigmatization [57].

AI provides a wide range of applications capable of supporting mental health training. A popular utilization of AI includes chatbots, also known as conversational agents, which are special human–machine interactive interfaces that allow a computer program to maintain a meaningful text- or speech-based dialogue with human users [27]. AI chatbots can teach users new skills, and provide them with guidance, support, and positive feedback during training. AI chatbots can increase users’ motivation for engagement in tasks that aim to improve mental health [57]. In addition, they can work as mood trackers, mental health assessment tools, and a self-management strategies tank [57]. The conversational agents can effectively imitate a therapeutic conversational style, allowing an interaction similar to a therapeutic conversation. Chatbots have the potential to encourage self-disclosure, a process in which a person is open to revealing personal information and interacting with others [58]. AI chatbots can be beneficial for people who deal with geographical constraints or dislike in-person interventions. Research has already revealed promising outcomes after using chatbots in interventions within a psychotherapeutic context [59].

ChatGPT is an advanced language model that makes use of deep learning techniques as a means to create human-like responses to natural language inputs [60]. ChatGPT can provide suitable and contextually relevant replies to a wide range of cues because it can capture the subtleties and complexity of human language. Its effectiveness depends on its capacity to elicit responses akin to those of a person, comprehend natural language, and adjust to various situations. ChatGPT can analyze significant amounts of knowledge, make interdisciplinary connections between various theories (i.e., mindfulness theories or psychological theories), and then provide users with the most relevant and updated knowledge [61]. A significant application of ChatGPT concerns the employment of personal assistants that can effectively assist users in dealing with mental health difficulties [60]. In addition, ChatGPT can also assist trainees by providing reminders, instructions, and information about potential risks. ChatGPT can also help therapists generate automated summaries of trainees’ interactions and medical histories as well as extract relevant information from records [60].

#### 3.2.2. The Potential Benefits from Extended Reality (VR, AR, MR, and Metaverse)

Extended reality (XR) refers to a wide range of immersive digital technologies where data can be displayed and projected. VR, AR, and MR are fundamental components of XR [62]. VR, AR, MR, and the metaverse concentrate on various features that make them promising technologies for training regulation skills in populations with mental health training needs.

VR refers to a computer-generated virtual environment wherein the subject can be immersed with the use of a VR headset and interact [63]. VR technologies provide controllable and personalized training environments [64,65]. Visual and auditory cues can be adjusted according to training objectives as well as the trainees’ needs. Distractive or stressful stimuli can be isolated, allowing users’ attention to work more flexibly. Positive and vivid visual cues as well as relaxing auditory cues can induce a state of calmness which in turn can accelerate training outcomes. Training can be safe for all, especially for people with high-risk behaviors or social anxiety [66].

Immersive technologies can make users feel fully present within the VR environment [65]. Presence constitutes a key training component in almost every mindfulness practice [67]. Moreover, VR technologies can provide users with new, vivid, and even transcendental experiences, encouraging them to observe and reflect upon these experiences as well as the mental and emotional states that these experiences provoke [68].

VR gives access to a powerful tool for mindful self-regulation that is none other than attention. Within the VR environment, users are familiarized with the functions of attention and gradually become aware of its regulatory power [69,70].

Mindfulness practices require trainees to develop sensory awareness. VR input/output devices, and visual, auditory, and haptic displays create extraordinary sensory experiences. In addition, these devices provide users with the unique opportunity to be aware of and manage sensory flow [69,71].

Mindfulness practices intend to provide users with positive experiences to induce those states of mind that permit users to effortlessly apply self-regulation strategies. VR can provide users with positive emotional cues either explicitly or implicitly [72]. In addition, VR can be combined with gamification techniques to induce one of the most beneficial states of mind known as the flow state [73].

While VR refers to a completely virtual environment, AR combines real and virtual objects. More specifically, AR describes a real-time view of a physical real-world setting that has been enhanced by the incorporation of virtual computer-generated information [74]. AR offers an interactive training environment with the potential to distract users from internal dysfunctional thoughts without losing contact with physical reality [75]. AR can help users with low mental imagery skills to make positive visualizations. All sensations can be increased with AR, facilitating mindfulness practice, especially for people with sensory difficulties. AR apps are accessible and user-friendly since a smart device is enough without the need for expensive equipment. Users can also add brain-sensing headbands as well as VR headsets [62].

MR is placed on the virtuality continuum in the middle of AR and increased virtuality. This environment integrates the real and virtual worlds in such a way that a bridge is created between them [63]. MR is considered a hybrid of AR and VR that enables users to physically engage with virtual objects in real settings [62].

The metaverse refers to the post-reality universe, a perpetual and persistent multiuser ecosystem blending physical reality with digital virtuality. It is based on the integration of technologies that allow multisensory interactions with virtual environments, digital items, and humans such as VR and AR. Τhus, the metaverse describes a networked and socially interactive environment that exists on perpetual multiuser systems. It enables real-time, seamless embodied user interaction with digital artifacts as well as ever-changing interactions with them [76].

XR (VR, AR, MR, metaverse) is increasingly being used in the field of mindfulness, but the effectiveness of VR-based mindfulness interventions in mental and emotional regulation remains unclear.

#### 3.2.3. The Potential Benefits of Non-Invasive Neurofeedback Technologies

The brain can pulse at a wide range of frequencies with each frequency level reliably reflecting measurable mental or emotional states. BCIs refer to computer-based systems that directly track, process, or analyze brain-specific neuro-data and translate these data into outputs [77]. The recorded activity is provided to users with real-time visual or auditory signals (feedback) corresponding to the levels of their concentration or/and relaxation [78]. The techniques to measure brain signals are divided into the following three categories: non-invasive, semi-invasive, and invasive [77]. The current study focuses on non-invasive BCIs that include sensors placed on the scalp to measure the electrical potentials produced by the brain (EEG) or the magnetic field (MEG).

Neurofeedback technologies allow users to be actively engaged in training procedures by monitoring how thoughts, emotional states, and brain activity interact and influence one another. By monitoring, analyzing, and understanding the feedback from brain activity and its impact on various aspects of human existence, users can be gradually trained to utilize this feedback as a guide to induce the desired mental or emotional state and effectively maintain this state [79]. With systematic exposure to this kind of mental training, the trainees become more adaptive when it comes to shifting out of rigid states indissolubly linked to mental health problems [78]. It is not by accident that neurofeedback technologies have been effectively utilized in various interventions to treat a wide variety of disorders such as attention deficit and hyperactivity disorder (ADHD), anxiety, depression, epilepsy, insomnia, drug addiction, schizophrenia, learning disabilities, dyslexia and dyscalculia, and autistic spectrum disorder (ASD), as well as other applications such as in the improvement in musical and athletic performance [80,81,82].

Smart headbands are non-invasive wearable devices that measure the users’ brainwaves, via electroencephalography sensors, and collect data and feedback in smartphones or virtual worlds to help users raise awareness of their mental and emotional state, the levels of their attention and anxiety, and generally their readiness to be effectively engaged in training tasks [83]. These neurofeedback technologies are non-invasive, portable, user-friendly, and low-cost devices. The devices are simple to set up and connect to a freely downloadable application on the user’s smartphone [34,84]. These devices can be used both in assessment and intervention, providing individualized solutions according to the trainees’ needs [78]. The feedback provided can foster self-reflection processes [85].

Mindfulness and neurofeedback share a common ground as both aim to train mental states and have independently been revealed to be effective in the treatment of a variety of mental health problems [78]. In addition, recent research has revealed that mindfulness practices can have an immediate and visible impact on practitioners’ brainwaves, opening a new avenue for research regarding the influence of mindfulness training on brain activity [86]. A growing number of studies outline that mindfulness practices can become even more accessible by EEG biofeedback technologies because of the immediate, real-time feedback that may help trainees to better focus attention and achieve a relaxed state of mind [32,78]. Researchers also mention that neurofeedback facilitates the implementation of mindfulness techniques aiming to train discrete brain regions [87]. Other studies support the idea that neurofeedback-assisted mindfulness training can help trainees enter those states of consciousness that make humans develop higher mental abilities [88,89].

In previous decades, researchers have explored the impact of meditation on the brain using EEGs and fMRI scans. However, the research was limited to labs and hospitals, because the equipment was too large and expensive for personal utilization. Nowadays, people are provided portable, easy-to-use, meditator-friendly EEG headbands [89].

**Figure 3:**
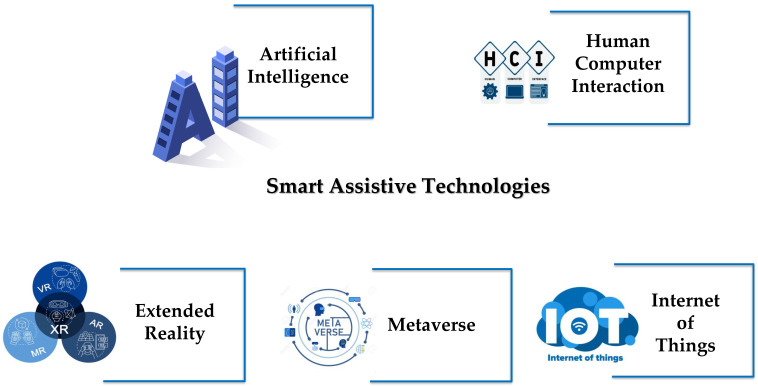
The top trending smart technologies as assistive tools in mental health training programs.

The international literature indicated that although smart technologies concentrate on a wide range of features that may be beneficial in mindfulness training, there is a need to gather, summarize, and synthesize existing evidence about the effectiveness of smart technologies on mindfulness training with a special focus on their contribution to training the self-regulation skills needs for mental and emotional balance [35]. Figure 3 presents the top trending technologies for assisting mental health intervention with a special focus on mindfulness training.

## 4. Results

### 4.1. Study Selection

Once all search terms have been combined and after applying all relevant limits, a final number of 1408 records were identified. A total of 352 studies were removed, because they appeared more than once in our results. Afterward, we evaluated the titles and the abstracts of the 1056 remaining studies, and a total of 658 studies were removed. Before full-text screening, we tried to retrieve the full-text PDFs. However, a total of thirty-nine studies could not be retrieved (i.e., not accessible). The remaining 359 studies followed the process of full-text screening. After an in-depth assessment of the studies, we decided to exclude 298 papers because of methodological issues (i.e., no relevant study design) or divergence from the predefined inclusion criteria (no relevant intervention or outcome).

**Figure 4:**
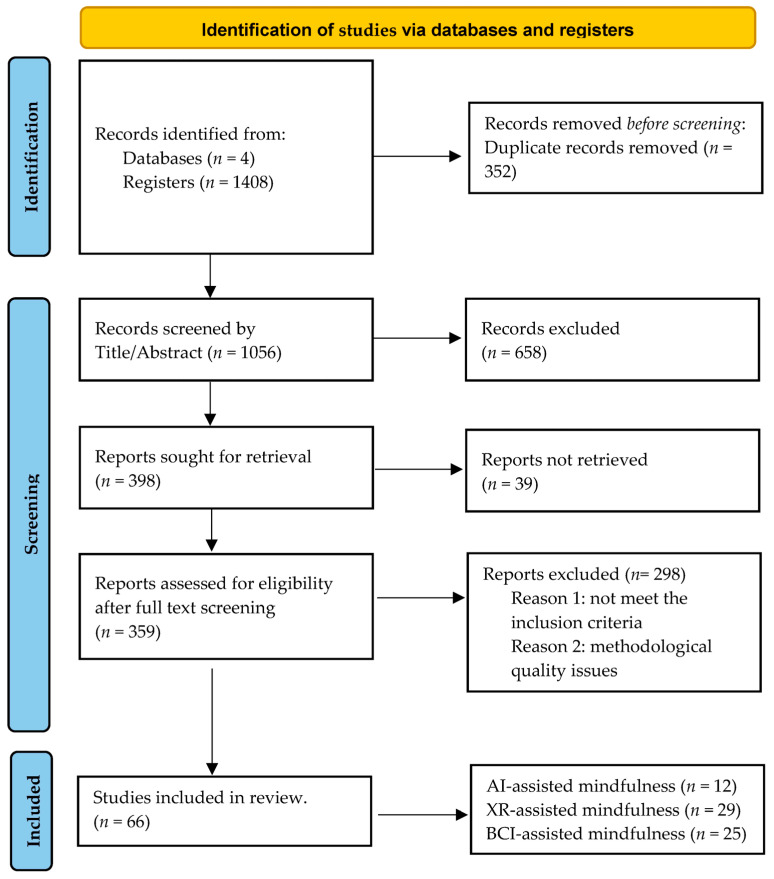
The PRISMA flow diagram.

The screening process led to a total of 66 studies for inclusion in the current review. The papers were classified into three homogenous groups. We selected twelve studies focused on AI-assisted mindfulness interventions. Twenty-nine studies were chosen concerning XR-assisted technologies, and twenty-five studies concerned the employment of BCI-assisted mindfulness and biofeedback interventions for the training of self-regulation skills among people with clinical and non-clinical conditions. Figure 4 illustrates the general screening procedures and the flow of selecting representative research.

### 4.2. Study Characteristics

The 66 selected studies utilized the data from a total of 21,432 participants. AI-assisted mindfulness training was evaluated in a sample of 18,991 participants. XR-assisted technologies were tested in a total of 1393 participants and a sample of 1048 subjects participated in mindfulness training supported by neurofeedback and biofeedback technologies. The majority of the participants were adults with a mean age of thirty years. The youngest participants had a mean age of 9.92, while the oldest participants were over 60 years old.

The collected data revealed that at least 5117 women took part in the digitally assisted mindfulness interventions (4338 with AI tools, 462 with XR technologies, and 317 with BCI and biofeedback tools), whereas a total of 1821 participants were male (1117 with AI tools, 481 with XR technologies, and 223 with BCI and biofeedback tools). One hundred and sixty-one participants were characterized as non-binary. In several studies such as those that used AI platforms for mindfulness training, researchers were not allowed to collect information about gender. Thus, it is not possible to shape an accurate picture of the gender of the participants.

The majority of the interventions (39/66 studies, 59%) were applied in samples with healthy subjects, while 40.9% of the selected studies examined populations with clinical symptoms. Among the clinical conditions that were mostly identified were the following conditions: neurodevelopmental disorders including ADHD and ASD. A significant percentage of the studies examined the effectiveness of digitally assisted mindfulness interventions in participants with anxiety disorders (i.e., generalized anxiety disorder, post-traumatic anxiety disorder), depressive disorder, panic disorder, and phobias and obsessions. It is noteworthy that mindfulness training assisted by AI was examined only in healthy subjects, whereas XR-assisted mindfulness was evaluated mostly in clinical populations. This is an interesting observation. We can assume that AI-powered interventions in mental health interventions are in their early stages. Such interventions among people with disabilities or mental health problems require the collaboration of professionals from different fields, as well as long-term research to adapt the design to the special needs of the users taking into account various ethical limitations.

**Figure 5:**
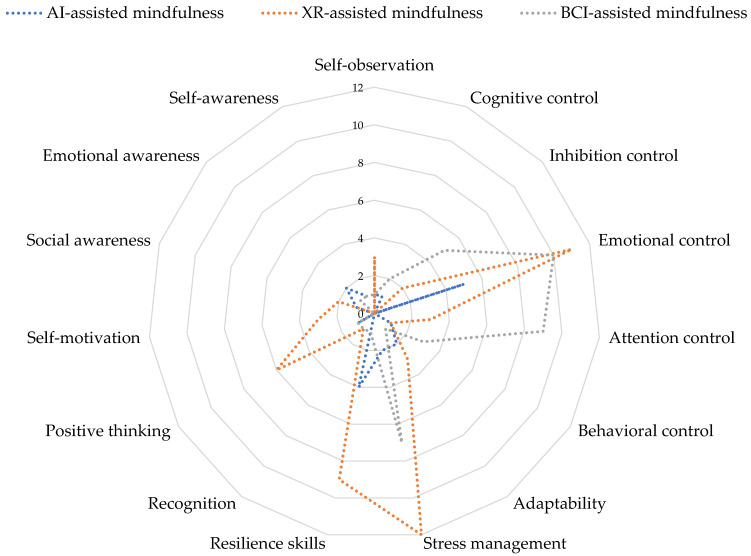
The central domains of self-regulation development according to the selected studies.

The regulation meta-skills that were mostly trained are the following: attention regulation, impulse control, cognitive control, and emotional regulation. In addition, special emphasis was given to resilience and stress management skills. Moreover, the researchers tried to raise participants’ self- and emotional awareness to encourage regulation. The participants were also trained to develop introspection and adaptation skills. The induction of positive feelings and positive attitudes was also the main component of the self-regulation training. Table 3 and Figure 5 present the most frequent self-regulation skills identified in the selected studies.

With regard to the type of studies, 37 studies from a total of 66 studies (56%) were randomized controlled trials, whereas the rest (*n* = 29) were experimental but not non-randomized.

The duration of the digitally assisted interventions lay between one session and 16 weeks. The average duration was 6 weeks.

**Figure 6:**
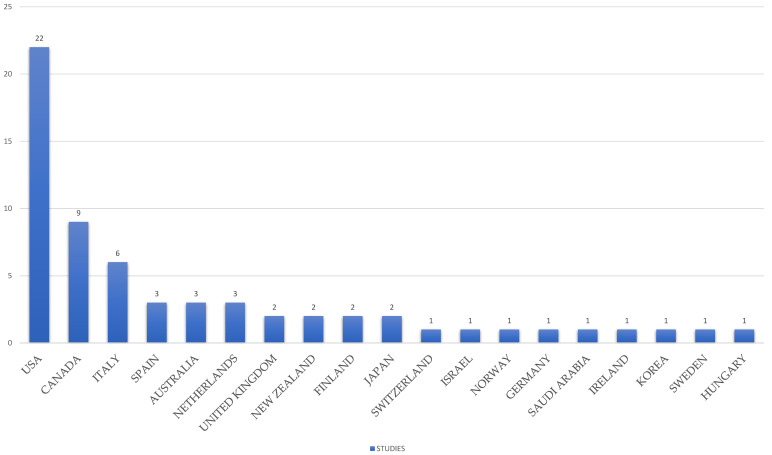
The number of studies per country.

Studies were conducted in the USA (*n* = 22), Canada (*n* = 9), Italy (*n* = 6), Spain (*n* = 3), Australia (*n* = 3), the Netherlands (*n* = 3), the UK (*n*= 2), New Zealand (*n* = 2), Finland (*n* = 2), Japan (*n* = 2), Switzerland (*n*=1), Israel (*n*=1), Norway (*n* = 1), Germany (*n* = 1), Saudi Arabia (*n* = 1), Ireland (*n* = 1), Korea (*n* = 1), Sweden (*n* = 1), Hungary (*n* = 1), China (*n* = 1), Turkey (*n* = 1), and Denmark (*n* = 1). Figure 6 presents the distribution of the countries that participated in relevant research.

**Figure 7:**
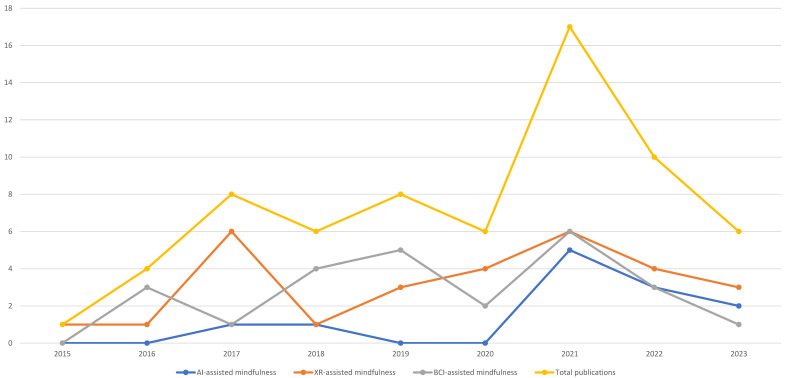
The production of studies between the years 2015 and 2023 (August).

We identified 66, relevant to our objectives, studies from 2015 to August 2023. The year 2015 was the least productive year, whereas the year 2021 was the most productive. We can assume that this is primarily due to COVID and the greater health needs and reliance on technology due to isolation. In August 2023, we ended our research. Thus, the number of studies may have increased in 2023. In general, it was observed that there has been an increasing trend in the employment of digitally assistive mindfulness intervention across the last ten years. Figure 7 presents the distribution of the studies per year from 2015 to 2023 (August).

### 4.3. Quality of Studies

The assessment of the selected studies’ quality indicated that the majority of the studies were consistent with the high-quality standards required for being included in the current review. Specifically, from a total of 37 randomized controlled trials, 32 had a low risk of bias, whereas one study had a high risk of bias. The main concerns were about the selection of the participants and the randomization, as well as the selection of the reported outcomes. The small sample size was another factor that influenced our decisions. The reviewers concluded that they had some concerns about four studies. With regard to the remaining 29 non-randomized studies, the assessment indicated 19 low-risk studies, 7 studies with a moderate risk, and 3 studies with a serious risk.

### 4.4. Smart Technologies in Mindfulness for Self-Regulation Training in Non-Clinical and Clinical Populations: The Main Findings

#### 4.4.1. AI-Assisted Mindfulness Training

AI-powered mindfulness apps that followed an emotional intelligence curriculum were found beneficial for raising social and emotional awareness and increasing resilience and emotional regulation skills among students. *SERMO* is an AI mobile application with an integrated chatbot that was effectively implemented in mindfulness training in combination with cognitive behavior therapy to support people with mental health problems in regulating emotions and dealing with thoughts and feelings [104]. *Ajivar* is an AI conversation platform that employs AI and machine learning to deliver personalized mindfulness training for training emotional intelligence skills using text-based conversations, videos, and related self-help techniques (i.e., positive affirmations). *Ajivar* can effectively respond to users’ emotions. At the same time, it can identify users’ underlying beliefs and appropriately adjust responses. Sturgill et al. (2021) [105] recruited a total of 99 college students and divided them into two groups. Fifty participants in the intervention group took part in the AI mindfulness training with *Ajivar*, while the remaining 49 students in the control group followed routine mental well-being instructions. The findings revealed that the AI platform acted as a mindfulness coach and significantly helped participants increase self-esteem, social and emotional awareness, and emotional regulation ability. In addition, anxiety and depression scores were significantly reduced.

*Wysa* is an empathy-driven, conversational AI agent for delivering digital mindfulness training. Specifically, the app through written and structured conversations can respond to users’ emotions and suggest therapeutic self-regulation strategies based on mindfulness, cognitive behavioral therapy, and positive psychology techniques. Inkster et al. [36] investigated whether *Wysa* could help 129 users develop self-management skills. The results indicated that mindfulness training with *Wysa* is effective and acceptable. The findings also revealed that *Wysa* significantly helped users develop positive self-expression as well as emotional resilience skills. However, researchers concluded that a more robust design is needed. *Wysa* was also assessed by Leo et al. [145] in a sample of 61 patients who experienced symptoms of pain, anxiety, and depression. The pain was reduced, while the scores in anxiety and depression rates indicated improved self-regulation abilities. Marcuzzi et al. [121] also confirmed with a randomized controlled trial that AI-based self-management apps employing mindfulness strategies can help people to regulate not only pain but also the subjective feeling of pain.

The selected studies indicated that AI-based mindfulness training can be effective for dealing with anxiety and emotional disorders. *Youper* is an AI chatbot designed to train users to monitor, recognize, and manage their thoughts and feelings. This AI app employs mindfulness training combined with cognitive behavioral therapy (CBT) and acceptance and commitment therapy (ACT). Mehta et al. [90] hypothesized that AI-based mindfulness training can help users to systematize training on regulating emotions just-in-time, which in turn would result in achieving long-term symptom reduction through the accumulation of these self-regulation successes. The data of 4517 users were analyzed based on longitudinal measures of anxiety and depression symptoms. After four weeks, users demonstrated improved emotional regulation skills, resulting in reduced anxiety and depression symptoms.

AI chatbots capable of delivering psychoeducation and self-regulation strategies based on cognitive behavioral therapy (CBT), positive psychology, and mindfulness techniques were found to be beneficial for training university students in self-control [106]. A proof-of-concept study evaluated the effectiveness of such an AI psychoeducational chatbot known as *Athena* in coping with stress using a sample of 71 university students. The results revealed that *Atena* significantly helped students to self-reflect on dysfunctional thoughts and feelings and to effectively employ self-regulation strategies in stressful situations. Self-awareness also increased, resulting in an improved self-management of anxiety symptoms. Participants also reported that they found the AI mindfulness chatbot an excellent way for a novice to start mindfulness practice.

Embodied conversational agents that teach positive life habits and suggest lifestyle modifications were found to help women living in rural areas adopt more positive and self-regulated lifestyles. Gardiner et al. [125] evaluated in a randomized controlled trial the effectiveness of an animated conversational character that simulates in-person interaction and delivers mindfulness training combined with suggestions on positive habits. The results demonstrated that, after one month, AI chatbots promoted behavioral changes including self-regulated behaviors. Potts et al. (2023) [139] evaluated a multilingual chatbot for improving mental health among people living in rural areas. The AI chatbot employed psychoeducational content and exercises such as mindfulness and breathing techniques, gratitude, and thought diaries in the chatbot. After 12 weeks of training, 348 participants significantly improved their psychological balance. Similar positive findings were revealed when AI chatbots were used in a sample of rural workers. The results indicated that 75 percent of the participants showed significant benefits [107].

*Kai.ai* is an AI-based personal companion that is utilized within an instant messenger app. *Kai.ai* allows users to choose how active they want to be. More specifically, users can either follow mindfulness instructions or be engaged in reflective conversations with the AI chatbot [126]. Vertsberger et al. [126] assessed the effectiveness of a mindfulness program that was based on Acceptance Commitment Therapy (ACT) and was delivered by the *Kai.ai* chatbot. A total sample of 10.387 adolescents were engaged with the conversational agent for an average of 45.39 days, showing significant improvements in mental and emotional balance. *Kao.ai* was also used in the research conducted by Naor et al. [140]. An analysis of 2909 users of kai.ai indicated significant improvements in their ability to maintain their psychological health balance.

**Figure 8:**
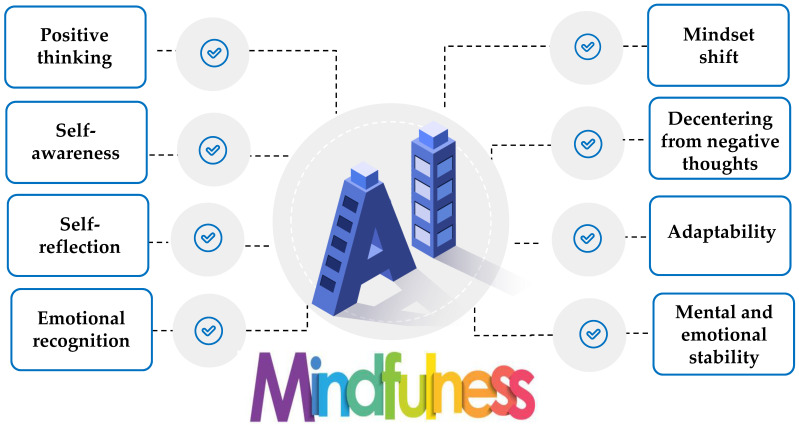
The core domains of regulation affected by AI-assisted mindfulness.

A summary of AI-assisted mindfulness training intervention is presented in Table A1 (Appendix A). Figure 8 presents the most frequent self-regulation skills identified in the selected studies.

#### 4.4.2. XR-Assisted (VR, AR, and MR) Mindfulness

VR, AR, and MR mindfulness were found to be beneficial for healthy young people, especially students, in developing the self-regulation skills needed for mental wellness, resilience, and academic achievement. For instance, a VR mindfulness program based on self-reflection practices was utilized in a sample of 280 university students who were divided into a VR mindfulness group, a conventional mindfulness training group, and a relaxation group. Six weeks later, the intervention group improved stress management, flexibility, emotional balance, and academic engagement [108].

Waller et al. [129] randomly allocated 82 undergraduate students into two guided mindfulness sessions, one VR and the other non-VR. Meditating in VR allowed students to experience positive feelings such as heightened connectedness that in turn increased their ability to regulate stress, distraction, and fatigue.

Kaplan-Rakowski et al. [131] investigated whether an immersive meditation intervention could be more beneficial than a video-based meditation in a sample of 61 students. VR-based meditation was found to be more or just as effective as video-based intervention.

Yildirim et al. [93] tested a brief VR-based mindfulness intervention in a sample of 15 young people and compared the results with an audio-based intervention and a control group. Participants in the VR-based intervention reported greater control over mind wandering than those in the guided audio condition, indicating that the immersive mindfulness intervention was more effective.

Other studies demonstrated that immersion and interaction provided by XR can increase curiosity and motivation which in turn facilitates self-regulation development. For instance, Roo et al. [141] evaluated in a sample of 12 subjects an MR system called “the inner garden” that utilized AR to assist mindfulness. It is about a multi-modal tangible artifact that took the shape of an AR sandbox. By shaping the sand, the users could create a living small world that was projected on the sand. The natural elements were connected to real-time physiological measurements to help the users remain focused. The users were also immersed inside the “garden” by using a VR headset. The results indicated that MR mindfulness increased curiosity and motivation and most importantly helped subjects to be better connected with their inner world. As a result, they could better manage external disturbances.

The results also indicated that VR in mindfulness training along with self-regulation can boost higher abilities related to creativity. Potts et al. 2019 [142] applied AR neurofeedback for meditative mixed reality in a total of 12 young healthy subjects. The results indicated that the immersion and interaction helped subjects to relax and develop creativity. In general, the studies indicated that VR could make mindfulness training less effortful and more effective for training self-regulation in novice practitioners [124].

The results indicated that VR-guided meditation can be an acceptable, feasible, safe, and cost-effective alternative health intervention for improving self-control in older adults. In a randomized controlled trial, Cinalioglu et al. [109] recruited 30 older adults aged ≥ 60 years with moderate anxiety. The intervention group (*n* = 15) received eight 15 min VR-guided meditation sessions. Eight sessions of VR-based mindfulness have been shown to help older adults increase relaxation and better self-regulate symptoms of anxiety and depression.

Studies also indicated that VR mindfulness can be an effective strategy for training workers at risk of burnout such as healthcare professionals. Tarrant et al. (2022) [110] investigated whether VR plus neurofeedback meditation can be effective in cultivating self-control skills among healthcare workers and compared the effects with a standard guided audio-only meditation. In contrast to the audio-only group, the VR group had significant gains in the measures of calmness and happiness. In a randomized controlled trial, Pascual et al. [132] evaluated a VR-based guided meditation intervention in the form of brief-paced breathing exercises using a sample of 32 healthcare professionals. The result demonstrated that VR-based guided meditation improved regulation skills without requiring an extensive time commitment for healthcare workers. The authors concluded that VR may be a more effective mindfulness platform for workers compared with standalone mobile meditation apps, especially when used regularly.

VR mindfulness training was found to be beneficial for the development of self-regulation skills among people with mild or severe anxiety disorder. Cikajlo et al. [94] investigated the effectiveness of group-based VR mindfulness assisted by virtual characters in anxiety disorders. Both the coach and the participants took part in the mindfulness program using their avatars. Eight weeks of training enhanced users’ attention regulation and stress self-management. Moreover, self-satisfaction was elevated. Jiang et al. [133] explored the feasibility of an AR mindfulness intervention on stress management using a sample of 10 healthy adults. The results of physiological measurements and the perceived stress questionnaire showed improvements in the regulation of stress.

Chavez et al. [134] recruited a sample of 30 young people at risk of anxiety and depression. They were randomized to participate for about 10 min in one of the following three interventions: (1) VR meditation, (2) audio meditation (via a web-based platform), or (3) VR imagery of historical pictures. Changes in anxiety levels and cortisol revealed that VR mindfulness could effectively help subjects control anxiety and depressive symptoms.

VR nature-based mindfulness combined with positive visualizations significantly improved the self-regulation capacity in post-traumatic stress disorder (PTSD). A sample of 96 young individuals received guided meditation with and without VR. In the VR condition, the participants could think more positively, and as a result, they were more able to control anxiety. It is noteworthy that participants reported that they preferred the VR condition [146].

Positive outcomes for anxiety disorder were found by Tarrant et al. (2018) [135] who evaluated a VR nature-based mindfulness program (*n* = 14) and compared the outcomes with a resting control condition (*n* = 12). The VR-based condition revealed better outcomes in self-regulation compared with the control condition. Electrophysiological markers also indicated reduced anxiety levels after the VR intervention.

VR mindfulness combined with other behavioral modification practices derived from Dialectical Behavior Therapy (DBT) was found to significantly help people with anxiety. A patient with anxiety took part in an immersive VR DBT^®^ mindfulness training program. The results indicated that the participant could think more positively and more flexibly manage negative feelings [111]. Similarly, forty-two subjects with generalized anxiety disorder were randomly assigned to either a conventional mindfulness intervention or VR-assisted DBT^®^ combined with mindfulness training. The results demonstrated that participants in the VR condition outperformed in terms of anxiety self-management, emotional control, and interoceptive awareness compared with the control group [147].

The results also indicated that subjects with phobias can significantly improve self-regulation competences after VR-assisted mindfulness training, especially when combined with other psychotherapeutic methods. Shiban et al. [112], for instance, combined VR, mindful breathing techniques, and exposure therapy. A total of twenty-nine subjects with phobias were randomly assigned to either the VR exposure treatment or VR exposure treatment with mindful breathing. The subjects participating in the VR condition significantly enhanced their self-control skills. Lacey et al. [113] combined mindfulness and cognitive behavior therapy with the assistance of a VR mobile app in a sample of 126 individuals with specific phobias. The use of VR combined with mindfulness and cognitive behavior therapy helped subjects to become more adaptive in the management of stressful memories. Seol et al. [136] employed a VR-based system designed to assist the mindfulness intervention in panic disorder. Participants in the training session reported that they could better stabilize their mood in stressful situations.

Several studies have focused on the effectiveness of VR mindfulness in populations with behavioral and emotional dysregulation. Navarro-Haro et al. (2016) [114] trained a woman with borderline personality disorder using a VR mindfulness DBT^®^ intervention. The woman, while immersed in VR, was asked to follow mindful instructions. For instance, she was asked to observe herself moving gently on a virtual river. After training, the woman was more able to control emotional distress and destructive thoughts of self-harm.

Habak et al. [66] recruited seventy-nine people with depression and examined the impact of delivering positive visualizations via a mixed-reality environment. The participants were immersed in extraordinary and relaxing landscapes. The immersion helped subjects to relax and think more positively. Moreover, they were found to inhibit negative thoughts and more flexibly manage emotions.

Veling et al. [115] investigated a VR mindfulness tool with immersive 360° nature-based videos in a total of fifty subjects diagnosed with anxiety disorder, depression, bipolar disorder, and psychosis. The experimental group followed a VR stress reduction mindfulness program, whereas the control group followed conventional relaxation practices. The experimental group was found to respond more positively, recognizing their responsibilities for dealing with challenges and being more ready to turn negative thoughts into positive ones.

VR mindfulness was found to effectively support the self-management of aggressive behaviors among people with depression and bipolar disorder by training users’ adaptive skills. Forty subjects utilized a VR calm room where mindfulness training (i.e., breathing exercises) took place, whereas the remaining twenty remained in a quiet room. The subjects interacted with the VR environment by gazing at chosen objects and, by using the hand-held controller, could adapt preferences and create individualized scenery. The experimental group was more able to relax and develop adaptive responses [127].

VR mindfulness was examined in populations with neurodevelopmental disorders such as ADHD and ASD, indicating positive effects in the training of self-regulation skills. Serra-Pla et al. [95] designed and tested VR mindfulness in a sample of fifty participants with ADHD. The intervention group (*n* = 25) participated in four 30 min mindfulness training with VR, while the remaining participants were treated only with psychostimulants. The authors concluded that VR mindfulness can significantly help people with ADHD to self-manage skills in populations with increased distraction and hyperactivity.

Mindful breathing exercises implemented with VR gaming technology and biofeedback were found to enhance self-regulation skills in a total of eight young people with ADHD. Players explored an underwater virtual world utilizing mindful breathing as a tool to control actions within the game. After six sessions, participants could better control anxiety and aggressive behaviors [122].

A virtual mindfulness intervention designed for group therapy was tested in a sample of thirty-seven adults with ASD. After six weeks of training, participants reported that they felt more self-controlled as they could better regulate anxiety and make use of self-control strategies. In addition, training in groups helped them to create social connections [130].

Finally, studies revealed that VR mindfulness can help people control feelings of pain [143]. Gromala et al. 2015 [143] described a VR mindfulness system designed to help patients with chronic pain regulate feelings of pain. By providing real-time visual and sonic feedback, the VR mindfulness system helped patients learn how to manage their pain.

**Figure 9:**
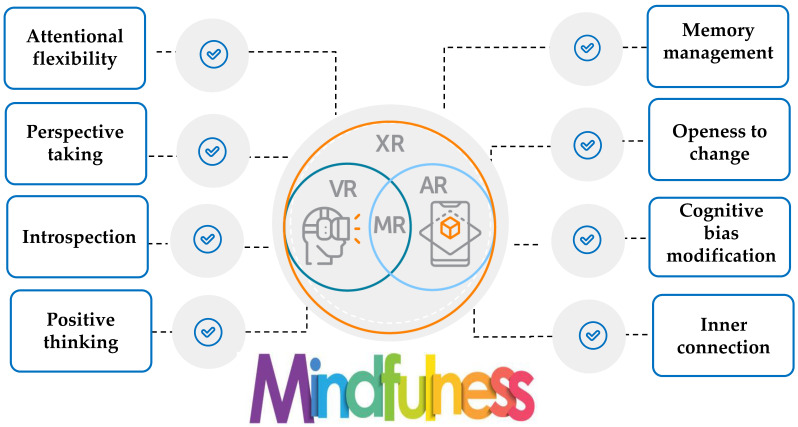
The core domains of regulation affected by XR-assisted mindfulness.

A summary of XR-assisted mindfulness training interventions is presented in Table A2 (Appendix A). Figure 9 presents the main contributions of XR-assisted mindfulness in the self-regulation mechanism.

#### 4.4.3. Non-Invasive BCIs and Biofeedback Mindfulness Interventions

The selected studies revealed that non-invasive brain-sensing wearable devices can significantly facilitate mindfulness training and encourage populations with no clinical symptoms to improve self-regulation skills, increasing the chances of mental and emotional well-being.

Studies revealed significant improvements in the ability to be aware and regulate crucial cognitive operations such as attention, after BCI-assisted mindfulness. For instance, Hunkin et al. [96] evaluated the efficacy of focused-attention meditation assisted by auditory EEG neurofeedback in a sample of 68 healthy adults. Neurofeedback was found to help trainees experience a state of increased awareness. The perceived control as well as attentional control, as measured by the Muse headbands, increased too. Crivelli et al. (2019) [97] investigated the potential of a BCI mindfulness training protocol in attention control, self-awareness, and psychological well-being. The experimental group practiced breathing exercises with the use of a wearable neurofeedback device, whereas the active control group engaged in conventional breathing practices. After fourteen sessions, both groups showed a significant reduction in response times and false alarms at computerized cognitive tasks, as well as a consistent improvement in the N2-event-related potential—all indicators of attention regulation ability.

Except for attentional control, studies indicated that the use of BCIs in mindfulness training helped subjects to better inhibit impulses and more flexibly control behavior when compared with conventional training. Balconi et al. (2019) [98], for instance, recruited fifty subjects and randomly divided them into an experimental and an active control group. Both groups followed mindfulness training with the difference that the intervention group used a wearable brain-sensing device, whereas the control group practiced conventional breathing exercises. The results showed that the experimental group outperformed in attention and behavioral regulation skills. Similar findings were presented in a randomized controlled trial conducted by Bhayee et al. [99]. An experimental group of 13 healthy adults followed a BCI mindfulness practice, while the active control group (*n* = 13) solved online math problems. The findings indicated that the intervention group improved attentional regulation as well as inhibition control.

BCIs were found to encourage subjects to raise emotional awareness, increase empathetic responses, and increase emotional regulation abilities [110,128]. In addition, subjects systematized an observational stance which in turn helped them to stabilize their mood more flexibly. Balconi et al. (2018) [13] evaluated the impact of a BCI mindfulness training intervention on healthy subjects’ ability to control mood states. Fifty-five participants were randomly divided into an experimental and an active control group. The experimental group received focused attention training using a wearable brain-sensing device. A smartphone app provided real-time feedback based on EEG markers. It was found that anxiety and mental fatigue were more effectively managed. Subjects were more able to balance mood. Specifically, they were more able to deal with negative emotions and adopt a positive perspective and a detached observational stance in challenging situations. Tarrant et al. (2022) [110] compared VR plus neurofeedback mindfulness training with conventional guided meditation. The results revealed that the experimental group could better control mood.

Mindfulness training with low-cost brain-sensing wearable devices was found to be a valuable tool for self-regulation, especially for employees whose professional position imposes elevated job duties. By way of illustration, a two-week mindfulness intervention assisted by a brain-sensing device was applied to a sample of sixteen professionals with top management duties. Electrophysiological markers of stress and neurocognitive efficiency, mood profile, and cognitive abilities measurements indicated that the BCI mindfulness group increased cognitive and emotional regulation skills. Mental fatigue was reduced and so were anxiety and anger. The authors concluded that BCI-assisted mindfulness could help employees become more productive in the workplace [91].

A significant percentage of the selected studies indicated that BCI-assisted mindfulness is feasible for young students to develop self-regulation skills and increase well-being and academic achievement. In a sample of 43 students, Kosunen et al. [100] evaluated a neuroadaptive meditation system that combined VR with neurofeedback. The results demonstrated that the feedback received helped students to increase attention control and regulate anxiety more effectively. In another study, Vekety et al. [35] evaluated an eight-session mindfulness stress reduction protocol assisted by EEG feedback on executive functions, attention regulation skills, and attention-related brain activity in a sample of thirty-one healthy elementary students. The intervention group showed significant improvements in inhibition as well as attention regulation skills. Brain activity demonstrated significant improvements, indicating increased calm/focused mental states and improved attentional flexibility.

Acabchuk et al. [117] recruited 53 university students who were novice meditators and divided them either into a meditation app group (*n* = 27) or a meditation app plus Muse neurofeedback device group (*n* = 26). Both groups demonstrated reduced distress and increased mindfulness scores following the mindfulness intervention. In another experimental study, Martinez et al. [123] randomized twenty students into a BCI-assisted mindfulness group (*n* = 10) and a control group. After 20 sessions, participants in the BCI mindfulness group were more able to perform self-regulated behaviors compared to the control group. Antle et al. [148] utilized a mindfulness-based neurofeedback system to teach self-regulation in a sample of twenty children living in challenging environments. A six-week BCI mindfulness intervention was effective in making children more capable of applying self-regulation strategies in real life. The results indicated that BCI-assisted mindfulness can be a self-regulation strategy not only for healthy populations but also for people with clinical symptoms. In a randomized control trial, Schuurmans et al. [137] applied a six-week BCI-assisted game-based mindfulness training in a sample of 77 adolescents with post-traumatic stress disorder. This study indicated a high potential for improving the ability to self-regulate anxiety symptoms. Crivelli et al. (2018) [92] investigated whether a four-week BCI mindfulness training program could help people with mild anxiety to increase cognitive control and neural efficiency. The experimental group received intensive mindfulness training using a non-invasive wearable EEG device supported by a smartphone, while the control group underwent breathing training. Electrophysiological indicators showed that the experimental group showed significantly improved markers of attention and executive control, suggesting improved attention regulation and self-control abilities.

BCI-assisted mindfulness was found beneficial for people with depression. An AR mindfulness training assisted by neurofeedback was applied to patients with depression. A total of forty-one adults were randomly assigned to either an AR mindfulness program assisted by frontal gamma asymmetry neurofeedback (*n* = 22), or an AR mindfulness program without neurofeedback (*n* = 19). The neurofeedback group outperformed in terms of engagement. Similar improvements were observed in mood regulation [116].

Promising findings were found in the case of obsessive-compulsive disorder (OCD). Richter et al. [103] evaluated a guided meditation assisted by a consumer-grade EEG-based biofeedback device in a sample of 55 subjects with OCD. The results indicated that subjects in the BCI-assisted mindfulness group (*n* = 27) were more able to inhibit obsessions and compulsiveness compared with the control group. Promising findings were also presented by Hawley et al. [101]. Seventy-one patients diagnosed with OCD received a meditation intervention via the use of a MuseTM device. EEG data, self-reports, as well as OCD measurements confirmed that the experimental group (*n* = 36) could better regulate obsessive behaviors and mind-wandering.

Two studies also indicated that BCI-assisted mindfulness may be feasible for people with cancer. In a randomized controlled trial, thirty patients with breast cancer were randomly assigned to either a BCI-assisted guided meditation or CD-based stress-reduction mindfulness. Measurements of stress, quality of life, and fatigue demonstrated an improved ability to regulate anxiety as well as a significant reduction in mental fatigue [84]. In a pilot study, Rolbiecki et al. [118] investigated whether VR nature-based mindfulness combined with neurofeedback could help fifteen patients with cancer to manage their mood and anxiety. The results indicated that BCI-assisted VR mindfulness helped subjects to better regulate anxiety and mood.

Except for EEG, other indicators of the physiological state such as pulse rate, respiration, heart rate variability, skin conductance, and blood volume were found to provide users with real-time audio, visual, or audiovisual feedback capable of facilitating self-regulation during mindfulness training [102]. This research also revealed relevant innovative tools that utilize wearable devices along with biofeedback technologies for providing personalized and interactive mindfulness training for self-regulation development. Can et al. [119] designed and evaluated an unobtrusive automatic stress management system with the use of consumer-grade smart bands. The system could monitor users’ stress levels. When a high stress level was detected, the system could suggest the most suitable mindfulness strategy taking into account the physical-activity-based contextual information. The 15 participants improved self-recognition skills. In addition, they could more flexibly regulate stress and intense emotions. Similarly, Shang et al. [138] evaluated an EEG-based mindfulness system that utilizes convolutional neural networks based on deep and machine learning methods to detect the state and trait effects of mindfulness training. Cheng et al. [144] presented the design and the initial assessment of a smart application called PAUSE that utilizes electrodermal activity as a means of interaction with the user. The mindful touch innovation significantly helped participants reach a relaxed state. Salehzadeh et al.’s [102] framework for interactive training makes use of the PAUSE app, which allows users to self-regulate their attention according to their abilities and conditions. EEG and other physiological markers indicated that personalized training tools can accelerate self-regulation skills. Heartmath is another inner balance mHealth app that utilizes heart-activity-based biofeedback (www.heartmath.com/ accessed on 21 November 2023). Minen et al. [120] conducted a randomized controlled trial with a total of 52 patients with migraine who had a history of anxiety and depression. After 8 weeks of training with the biofeedback Hearthmath app, the participants could better improve their anxiety and mood.

**Figure 10:**
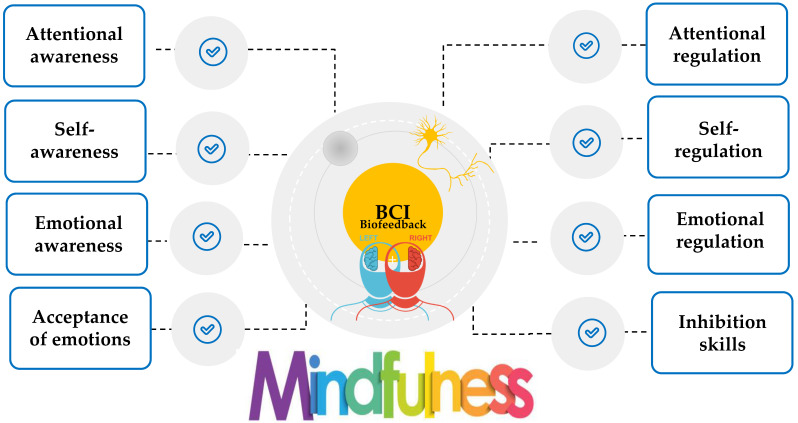
The core self-regulation skills trained during BCI-assisted mindfulness.

A summary of BCI-assisted mindfulness training interventions is presented in Table A3 (Appendix A). Figure 10 presents the main contributions of BCI-assisted mindfulness in self-regulation mechanism.

## 5. Discussion

### 5.1. AI-Assisted Mindfulness: Opportunities, Challenges, and Future Directions

In general, the results showed that AI applications effectively assisted mindfulness training, supporting users to become more conscious and self-regulated. Individuals who received AI mindfulness training were more able to think positively. The interaction with AI coaching assistants, conversational companions, digital screeners, and virtual therapists made them more reflective and flexible. They were more capable of decentering from negative thoughts and feelings and redirecting attention to a more positive perspective. As a result, users were more open to changing perspectives and behaviors, modifying dysfunctional thinking patterns, and adopting new and more functional habits. The results of the selected studies confirmed that AI not only made mindfulness training more accessible but also AI significantly assisted the training of the self-regulation skills needed for mental and emotional balance.

AI chatbots were found to be effective in teaching mindfulness strategies for self-regulation. The instruction was both direct and indirect. The AI coaching agents could explicitly provide advice and suggestions for self-regulation or develop reflective interactions with the users, intending to help them to decenter from dysfunctional thoughts or shift their mindset, become more adaptive, and perceive difficult situations from a different point of view [106,125].

The results indicated positive outcomes for a wide range of social groups including students, workers, citizens who live in stressful rural environments, and minorities [107]. In addition, the findings revealed positive results for populations with both clinical and nonclinical conditions. AI-assisted mindfulness chatbots were found to help people dealing with anxiety and mental and emotional disorders. By systematically engaging patients in the real-time monitoring, identification, and management of negative thoughts and feelings, AI chatbots helped people with mental health problems to develop the self-regulation skills needed for mental and emotional stability. The systematic repetition helped trainees to experience long-term symptom reduction through the accumulation of these self-regulation achievements [90,104]. It is noteworthy that most patients reported that they found their experience satisfactory. Both patients and researchers who took part in such interventions reported that they found digitally assisted mindfulness training supportive and useful [90].

The results revealed that AI conversation platforms that apply emotional intelligence curricula blended with mindfulness strategies can effectively train mental and emotional resilience skills and promote general psychological well-being. The AI-based emotionally intelligent mobile apps were very beneficial because they could identify users’ emotions and underlying beliefs and respond empathetically. In addition, AI chatbots delivered scalable mindfulness training with individualized content [106]. In addition, the feedback provided encouraged users’ self-reflection, maximizing self-regulation capacity [36,105].

The real-time feedback that AI-powered mindfulness interventions provided helped trainees to better realize personal strengths and weaknesses and to identify areas for self-development, and most importantly encouraged them to apply self-regulation strategies for mental and emotional balance. In the case of people with mental health issues, the results indicated that AI through personalization significantly helped participants to complete mindfulness programs and systematize practice in real-time situations.

Although AI technologies can minimize the barriers that face-to-face interventions may bring, their use should be complementary and under the supervision of healthcare professionals, especially in cases of severe mental health problems [36]. For instance, the utilization of remote digital screening in anonymous populations presents a significant challenge in the absence of in-person clinical interviews. However, several AI-powered mindfulness apps provide users the opportunity to be connected not only with a virtual assistant but also with a human coach to maximize accuracy and positive outcomes [36].

The use of AI as an intervention tool in mindfulness training may lead to ethical and legal problems [60]. Most studies that utilized personal data have outlined the urgent need to better protect privacy and increase data security [106]. The applications gather and analyze sensitive data that incorporate information about the users’ mental and emotional health. In that vein, data policies should be clearer and protect users from misuse of sensitive information.

It is essential to outline that ethical considerations do not concern only data privacy. For instance, smart systems can develop deceptive behaviors such as pretending to be human beings, having real-life stories, emotions, and memories. In addition, ethical concerns arise from the use of persuasive techniques to direct users’ decisions, the increased risk of overdependence, as well as the replacement of human relationships [149].

The ethics of using AI to modify users’ behavior is another issue of concern, especially in the case of vulnerable populations such as those with mental health problems. For that reason, the design of AI agents should take into account the special needs and characteristics of the target group to ensure it does not deliver harm. For instance, users with schizophrenia may feel that an AI system is surreptitiously monitoring them, intending to harm them [150]. As Bickmore et al. [150] outlined, AI tools should be appropriately designed to maximize therapeutic outcomes and minimize the risk of harm. Thus, this study is in line with studies highlighting that adaptation according to users’ needs must be a requirement [149,150].

Even though AI algorithms generate a lot of data-driven information, they might not be able to perceive the whole context of a person’s mental health background. In addition, AI is often unable to differentiate between reliable and unreliable sources. This may lead to inaccurate or biased results. In this light, AI autonomous mindfulness agents may not be safe enough for autonomous decision making without supervision from human professionals [60]. In mindfulness interventions that employed AI-powered technologies, such as chatbots, participants reported that they would prefer to have a more personalized and realistic interaction with AI conversational agents. In addition, they outlined that AI agents should be further developed to better understand the various subtleties of human mood. As they reported, AI-powered mindfulness applications lack the empathy and emotional support that human beings need as fuel to continue their effort [106].

Another risk concerns the over-dependence on AI assistance. Mindfulness training interventions primarily aim to make trainees more conscious, autonomous, and self-directed, capable of voluntarily regulating their mental powers and inducing those mental states that make them successful and satisfied. However, the over-reliance on smart technologies may undermine the intensity of trainees’ efforts. As stated by Deborah et al. 2023 [149], overdependence does not only reduce training outcomes but most importantly restricts users’ independence when making decisions. This is one of the most important ethical issues that determine the appropriateness of AI-powered technologies in mental health interventions.

The efficacy and acceptability of AI-powered mindfulness interventions for mental and emotional balance are promising. Studies showed that AI-assisted mindfulness has the potential to be applied to the self-management of anxiety and emotional disorders [90]. However, more experimental research with randomized controlled trials is needed to evaluate the effectiveness of such interventions in recruiting participants with mental or emotional disorders. Future research should also focus on designing AI-powered apps with higher accuracy and precision. This objective can be achieved only with the collaboration of engineers, psychologists, coaches, and other researchers for relevant fields [126].

In addition, it is essential to develop AI agents capable of providing empathetic listening, accurate interpretations, and responses. In addition, the inclusion of additional elements that foster interaction and engagement (i.e., meditation videos, graphics, and visuals) would significantly improve AI-powered mindfulness chatbots [106]. Moreover, improvements in AI systems in terms of dealing with unexpected user inputs and the variability in chatbot responses could significantly improve training outcomes [104]. Finally, the effective use of humor could make virtual coaches even more attractive.

Research has not yet provided experimental studies focusing on the effectiveness of ChatGPT as a tool for mindfulness training. ChatGPT has the potential to support mindfulness training by providing both trainers and trainees with relevant information about mindfulness training, answering questions, and creating various guided meditation scripts [60]. However, evidence-based research is needed. Future studies should further explore the potential of ChatGPT to be involved in such interventions as mindfulness and conduct randomized controlled trials in clinical and non-clinical settings.

**Figure 11:**
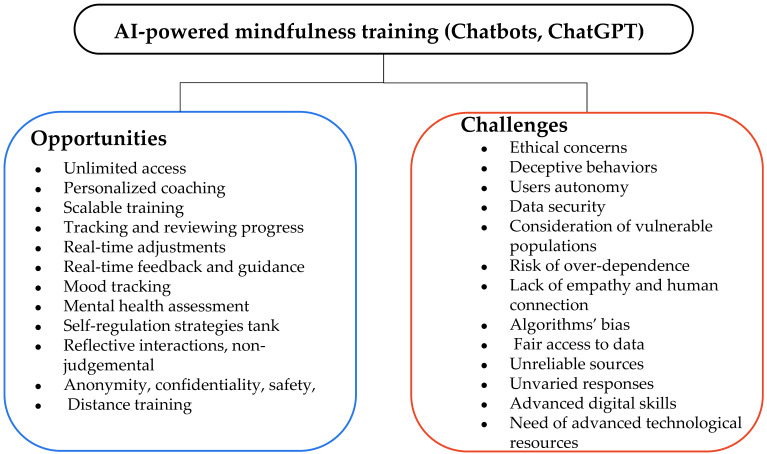
AΙ-powered mindfulness: opportunities and challenges.

Among the most significant limitations observed were the limited and unbalanced comparison group sizes and not being able to account for variables such as age and gender [36]. Figure 11 summarizes the main opportunities and challenges derived from the use of AI-powered technologies in mindfulness training.

### 5.2. XR Mindfulness Training: Opportunities, Challenges, and Future Directions

The results indicated that XR technologies including VR, AR, and MR have a significant potential to facilitate the implementation of a wide range of mindfulness strategies, providing fully vivid, interactive, reflective, and engaging training experiences. Most importantly, XR technologies helped subjects develop a better understanding of the unique power of the mind to reflect and regulate the physiological and neuropsychological mechanisms that are responsible for mental and emotional well-being. XR technologies helped subjects to better realize how mental states influence the current state of being and behaving as well as how XR mindfulness training can assist them to voluntarily induce functional states of thinking. In addition, they were allowed to employ mindfulness strategies more effectively for mental and emotional regulation. XR technologies in mindfulness training provided trainees with advanced VR experiences that, combined with mindfulness training, encouraged trainees to explore and expand the boundaries of self-regulation. In other words, XR mindfulness helped them to realize that they had more control than they ever thought.

The selected studies revealed that XR mindfulness can train self-regulation skills in both clinical and non-clinical populations, increasing the opportunities for sustainable living characterized by a self-directed, autonomous, and psychologically balanced life. In the non-clinical populations, VR, AR, and MR mindfulness helped subjects develop resilience skills including optimism, flexibility, patience, self-awareness, and self-confidence [143]. Workers at risk of burnout were found to significantly increase the above skills, indicating the importance of implementing such methods in places for increasing workers’ well-being and productivity [110,132]. In addition, typically developing students as well as students with special educational needs significantly increased a wide range of self-regulation skills [108,122,131]. Students were more able to concentrate, effectively manage exam anxiety, be self-confident, and achieve a state of emotional balance [108,131]. Finally, older people improved their self-regulation skills after mindfulness training assisted by VR [109].

VR, AR, and MR mindfulness were found to increase self-regulation capacity in populations with neurodevelopmental disorders, neuropsychiatric conditions, anxiety disorders, behavioral and emotional disorders, as well as intellectual disabilities [66,147]. It was revealed that these populations developed a wide range of regulation skills including attentional control, inhibition control, and emotional control. After training, subjects had an improved control over attentional operations which in turn helped them not only to improve mental abilities but also to better regulate the intensity of thoughts and feelings. Subjects were more able to change their point of view and to think more positively, strengthening their ability to deal with challenging situations. Thus, the improvements in self-regulation allowed a significant percentage of these populations to achieve reductions in hyperactivity, aggressiveness, depression, phobias, panic, and emotional instability [112,134].

VR, AR, and MR mindfulness interventions were found to be equally effective with traditional mindfulness training. In many cases, the assistance of XR technology led to better performance compared to other interventions. It is not by accident that participants expressed their preference for the use of VR [146].

VR facilitated the blending of mindfulness training with well-established psychological approaches (i.e., cognitive behavioral therapy and exposure therapy), accelerating positive outcomes [112,147]. In addition, XR technologies were found to fit well with biofeedback technologies and gamification elements, increasing engagement and providing more feedback for regulation [122]. VR technologies allowed group-based training to take place, assisting individuals with social anxiety to develop social connections and functional relationships. The use of virtual characters and virtual coaches significantly contributed to that end [94].

Mixed-reality platforms that allowed users to be physically involved with the physical environment whilst visually exploring the virtual world increased users’ sense of agency. High levels of presence in environments brimming with positive cues helped subjects regulate their feelings of hopelessness, balance their mood, and increase positive future thinking [66,141].

The current review deals with several limitations. In the selected studies, a limited number of challenges have been identified. Several participants reported that they felt uncomfortable with the head-mounted display. As a result, they were distracted with a reduced sense of presence [124]. In some cases, the short duration of the intervention, the lack of follow-ups, and the heterogeneous measurements made it more difficult for us to export accurate results.

**Figure 12:**
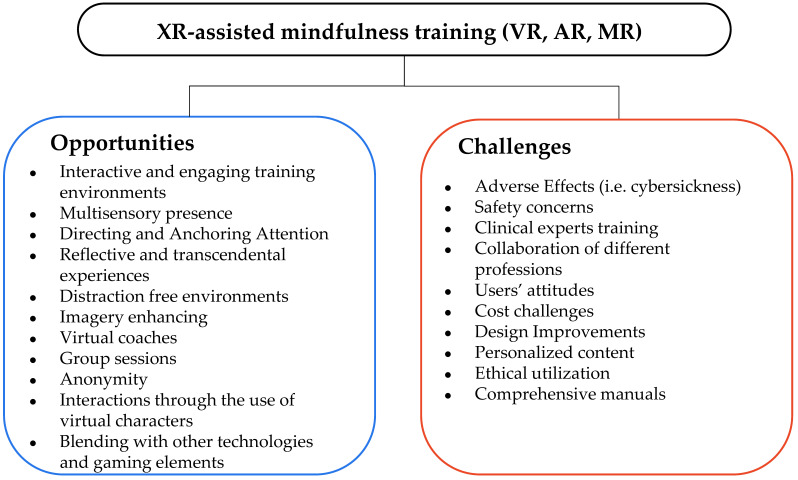
XR-assisted mindfulness: opportunities and challenges.

Last but not least, XR technologies, although providing unique advantages, can cause diverse effects such as cybersickness, including symptoms of fatigue, nausea, eye strain, and disorientation [151,152]. However, mindfulness practices can help trainees overcome various adverse effects [153]. Other challenges include the clinical experts’ training, the equipment’s cost, the users’ attitudes, the design of personalized training sessions, and the validation of XR applications [151]. Virtual and mixed reality should be regarded as an accessible platform for training individuals with mental health problems [66]. Figure 12 summarizes the main opportunities and challenges derived from the use of XR-assisted technologies in mindfulness training.

We suggest that XR-mindfulness should be used in educational settings to promote mental health and emotional well-being among typically developing students as well as students with special education needs. Except for young people, XR mindfulness can be effectively applied to workers, especially those who are at risk of burnout. Moreover, XR mindfulness can be used to assist the self-regulation training of older adults who are at risk of anxiety and depression.

### 5.3. BCI-Assisted Mindfulness Training: Opportunities, Challenges, and Future Directions

BCI-assisted mindfulness helped subjects to develop the metacognitive skills required for the self-management of cognition, emotion, and behavior, including self- and emotional regulation, self-monitoring, and attentional and inhibition control, which are well-recognized indicators of academic achievement, peak performance, and mental and emotional well-being. In addition, the increased awareness acquired over mental and emotional operations allowed trainees to flexibly apply self-regulation strategies each time they observed disturbances due to mental fatigue, anxiety, and intense emotions. Most importantly, trainees became more self-directed and independent.

Mindfulness training with the use of EEG feedback allowed subjects to voluntarily apply mindfulness strategies for mental and emotional regulation. Practitioners had the unique opportunity to receive feedback from data derived from non-conscious operations and utilize them as a tool for conscious self-regulation. Trainees, with the assistance of the neurofeedback, could voluntarily balance their brain activity, increasing for instance alpha waves that induce a state of relaxation and concentration and reducing beta waves that intensify mind-wandering and anxiety. By balancing brain activity, users improve the cognitive functions that play a self-regulatory role such as attention and inhibition control [99,103].

BCI assisted mental and emotional regulation in many ways. BCI-assisted mindfulness helped trainees learn how to voluntarily manage attention, which in turn allowed them to use the power of attention in the regulation of intense emotions [35,96,99,101]. Subjects through feedback were more able to perceive, recognize, and accept emotions. The employment of BCI mindfulness reduced mental fatigue, fostering self-control capacity [84]. Moreover, the engagement with neurofeedback increased the sense of agency, which in turn led to an improved ability for self-regulation [110,118].

The use of neurofeedback had a positive influence on emotional operations. The regulation of emotions was a less effortful task. In most studies, participants could think more positively and empathetically, deal with intense emotions, accept negative emotions, and in general, perceive events with a positive mindset [13,92,97].

BCI-assisted mindfulness raised trainees’ motivation for systematic practice and increased their engagement with self-regulation strategies during and after training sessions. It is noteworthy that trainees who received this BCI mindfulness were more able to transfer self-regulation abilities in real-life situations.

One limitation we can mention concerns the use of equipment. Most studies utilized low-cost consumer-grade EEG devices. In addition, several studies were conducted using a small sample of patients and measured the effectiveness of BCI mindfulness in a short period. Thus, future research should focus on advanced BCI systems and protocols for safer data. Moreover, future studies should explore the effectiveness of BCI-assisted mindfulness in larger and more diverse populations, while longer follow-up periods are needed to create a robust evidence base [101]. More research and the employment of more advanced signal processing tools are required to design neurofeedback-based mindfulness protocols that fit different objectives and the different training needs that various clinical populations may have.

It is essential to keep in mind that no intervention works for everyone. There are always potential dangers of adverse effects. Non-invasive brain-sensing wearable devices are considered safe enough because a limited number of adverse effects are mentioned. In most selected studies, researchers monitored participants for adverse effects. In most studies, no major adverse events were reported [84,117,148]. Some common side-effects of neurofeedback may include anxiety, emotional lability, irritability, fatigue, and mental fogginess [154].

**Figure 13:**
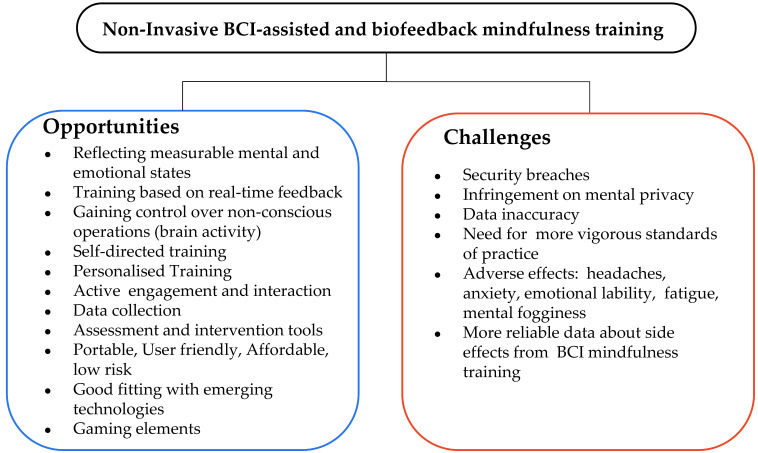
BCI-assisted mindfulness: opportunities and challenges.

It is also crucial to emphasize that, as with any therapeutic intervention, patients who seek neurofeedback for clinical purposes should take advice from licensed practitioners because inappropriate training can lead to adverse effects [154]. Moreover, the design of BCI-assisted mindfulness protocols should be appropriately designed to fit the target group’s characteristics and training needs. For instance, in the case of people with ADHD, brain activity is significantly disrupted. Thus, the researcher should select an appropriate mindfulness program along with a BCI protocol that fits the target group’s needs. For training to be effective and diverse effects minimized, it is essential to perform assessments to make the training personalized to the different brainwave patterns and symptoms of each participant, because each person may need training at different regions of the brain [155,156]. Figure 13 summarizes the main opportunities and challenges derived from the use of BCI-assisted technologies in mindfulness training.

BCI-assisted mindfulness training can be implemented in educational settings to equip students with the self-regulation skills needed for mental and emotional well-being [35]. In addition, BCI can offer personalized training for students with special education needs and disabilities. BCI mindfulness can be implemented in workplaces where employees are at risk of burnout. Employees would have more opportunities to manage anxiety and increase their problem-solving and decision-making capacity [85].

### 5.4. Final Considerations

The results of this study provided us with significant evidence about the positive impact of digital technologies in mindfulness training for the development of self-regulation skills. However, some studies provided evidence for a significant improvement [105,106,125,139,140,145], whereas other studies indicated that the intervention had a positive but not significant impact on self-regulation or that more research with larger samples and longer periods of training was needed [36]. None of the selected studies showed that the employment of digitally assisted mindfulness harmed self-regulation. Other studies mentioned that the positive effects were not maintained for a long period [90]. Hunkin et al. [96] mentioned that different aspects of feedback may be either helpful or unhelpful. In addition, several researchers mentioned some adverse effects that may indicate risks for users, especially for vulnerable groups such as those with mental health problems and disabilities. Several participants reported that the use of the head-mounted display did not allow them to concentrate and improve self-control skills [124]. The researchers outlined that digital technologies can improve the training. However, special focus should be given to the design. For instance, people with schizophrenia may feel that an AI system is surreptitiously monitoring them, intending to harm them. Thus, the AI-powered intervention, for instance, may have the opposite results [150]. Although the results of the current systematic review demonstrate the positive effects of digitally assisted mindfulness training in self-regulation, special focus must be given to the risks and challenges derived from the use of technologies. It is important to keep in mind that technology is neither positive nor negative. The ways of use determine the technologies’ impact on human health and behavior. Thus, special focus should be given to studies that outline the need to be aware of the possible risks derived from the use of digital tools [150]. Especially, ethical issues should be considered first [149,150].

We conclude that digital technologies can positively assist mindfulness training in the following ways: (a) providing explicit suggestions and coping strategies; (b) promoting reflection and interaction; (c) decentering users from unhelpful stimuli; (d) motivating, engaging, and rewarding users; (e) providing accurate and real-time feedback; (f) alerting users; (g) providing scalable training; (h) being user-friendly; (i) being designed according to well-established theories; (j) being adaptive according to users’ needs; (k) being personalized; and (l) being ethical. However, it essential to outline that each technology has special features and different strengths and weaknesses. In addition, the special characteristics of the participants and the objectives can determine to a significant extent the appropriateness of each type of technology. For instance, people with autism may not accept head-mounted displays. However, additional research is needed to find out in which cases each technology works better.

**Figure 14:**
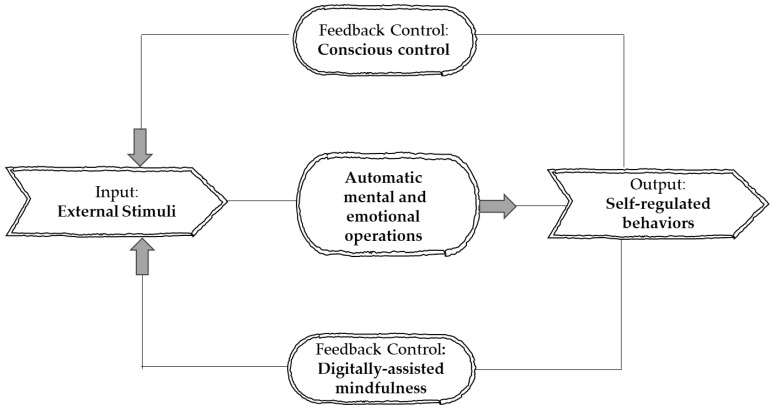
Digitally assisted mindfulness and metacognitive control allowed subjects to effectively manage dysfunctional thoughts and emotions, allowing the externalization of self-regulated behaviors.

Looking at the results of this review, we conclude that mindfulness training assisted by digital technologies helped participants to increase their potential for conscious control over automatic responses and, as a result, to improve their ability to initiate self-regulated behaviors (Figure 14). Specifically, external stimuli, especially stressful stimuli, tend to activate automatic (non-conscious and thus uncontrollable) mental and emotional reactions that cause internal imbalance and are responsible for the dysregulation of behavior. Mindfulness training assisted by digital technologies joins hands with self-control operations and strengthens users’ conscious control skills, allowing them to effectively manage stressful stimuli, inhibit impulses, achieve inner balance, and, finally, externalize self-regulated behaviors (Figure 14).

## 6. Conclusions

In the aftermath of the global pandemic, the need for digitally assisted mental health interventions, including mindfulness training, is expected to upsurge. Given this fact, the current study aimed to investigate whether smart technologies can be safe, feasible, and effective assistive tools in mindfulness training. In addition, we co-examined the potential of smart technologies to support the training of self-regulation skills in both clinical and non-clinical populations. This review paper concludes that smart technologies are feasible and safe enough to effectively assist mindfulness interventions, having a significant potential to train a wide range of self-regulation skills, which are considered the gateway to mental and emotional well-being.

Mindfulness training assisted by smart technologies effectively met trainees’ psychological needs, encouraging them to internalize motivation for training and less effortfully develop self-regulation skills. Smart technologies were found to increase participants’ willingness to continue efforts to improve their ability to regulate themselves.

The implementation of mindfulness training combined with AI and IoT applications, immersive technologies, and wearable technologies, including brain-sensing headbands, made digital inclusive education even more effective. The use of smart mindfulness training can be an innovative, accessible, and cost-effective solution for educational settings to help students, teachers, and caregivers become more resilient, self-regulated, creative, and innovative.

Results demonstrated that digitally assisted mindfulness training can help employees to better deal with job-related challenges and increase dedication, confidence, and productivity. In that vein, such training opportunities can be an effective avenue for creating sustainable working environments.

Smart technologies can support remote mindfulness training, encouraging users to take care of their mental health from anywhere and at any time without losing the benefits of safety and supervision from healthcare professionals. Smart technologies can also help trainers design and implement more flexible mindfulness interventions that address a wider range of participants with different training needs.

Smart technologies in mindfulness training should be utilized, taking into consideration ethical and legal guidelines, ensuring safety and accuracy, protecting privacy, and preventing bias [157]. The overuse of digital aids is another risk that may reduce the intensity of effort [92]. In any case, designers, trainers, and trainees need to be aware of both the challenges and opportunities derived from the emerging technologies used in mindfulness training [158].

Developing effective smart mindfulness training programs should be a priority in the coming years with the additional view of increasing accessibility to high-quality well-being programs. However, it is essential to provide equal access to the resources or smart technology needed for the implementation of digitally assisted mindfulness training.

Digitally assisted interventions could help future societies deal with the increasing percentage of mental health problems as well as the resulting economic costs more effectively [126]. As digital technologies become smarter and less biased, the benefits of mindfulness training in mental health are expected to increase.

## Figures and Tables

**Table 1:** The inclusion and exclusion criteria according to which studies were selected.

Inclusion	Exclusion
(a) Experimental studies (i.e., randomized controlled trials). (b) Published after 2010. (c) Mindfulness was the primary type of intervention with the assistance of smart devices. (d) Studies evaluated aspects of self-regulation. (e) Participants with clinical and no clinical health conditions.	(a) Systematic reviews, meta-analyses, and book chapters were not included. (b) Protocols and design frameworks without testing the feasibility of the proposed intervention. (c) Studies that investigated the effectiveness of mindfulness interventions without the assistance of smart technologies.

**Table 2:** The main search terms used in four databases.

The Main Searching Keywords in Search Strings
“Mindfulness” OR “meditation” OR “mindful breathing” OR “guided meditation” OR “open monitoring” OR “focused attention” OR “guided imagery”
AND
“Smart technologies” OR “artificial intelligence” OR “conversational agents” OR “immersive technologies” OR “virtual reality” OR “virtual companions” OR “augmented reality” OR “mixed reality” OR “brain-computer interfaces” OR “biofeedback” OR “metaverse” OR “chatGPT”
AND
“self-regulation” OR “emotional regulation” OR “self-awareness” OR “emotional awareness” OR “emotional recognition” OR “impulse control” OR “attentional regulation” OR “adaptability” OR “stress management”

**Table 3:** The most frequent self-regulation skills developed after digitally assisted mindfulness.

Domains of Self-Regulation	AI-Assisted Mindfulness	XR-Assisted Mindfulness	BCI-Assisted Mindfulness
Cognitive control	[90]		[91,92]
Attention control		[93,94,95]	[35,92,96,97,98,99,100,101,102]
Impulse control		[66,95]	[35,92,99,101,103]
Emotional control	[90,104,105,106,107]	[66,94,108,109,110,111,112,113,114,115,116]	[13,84,91,102,110,116,117,118,119,120]
Behavioral control	[121]	[122]	[92,98,123]
Introspection	[106]	[114,115,124]	[13]
Flexibility	[125,126]	[108,113,127]	[35]
Recognition		[115]	[119]
Emotional awareness	[105,106]		[128]
Self-awareness	[106]		[96]
Social awareness	[105]	[129,130]	
Stress management	[105,125]	[114,122,127,129,130,131,132,133,134,135,136]	[100,117,118,120,137,138]
Resilience	[36,105,139,140]	[94,108,109,110,127,136,141,142,143]	[144]
Positive thinking	[36]	[66,94,111,113,115,129]	[99]
Self-motivation		[108,110,113]	

## Data Availability

All data relevant to the study are included in the article or uploaded as Supplemental Information.

## Supplementary Materials

The following supporting information can be downloaded at https://www.mdpi.com/article/10.3390/bs13121008/s1. The Prisma 2020 checklist is included as Supplementary Material [38].

## Appendix A

**Table A1:** Summary of AI-assisted mindfulness training interventions.

Reference	Country	Digital Design	Mindfulness Program	Sample	Clinical Condition	Duration	Type of Measurement	Research Design	Main Improvements
Gardiner et al., 2017 [125]	USA	Embodied conversational agent	MBSR	*n =* 61, (*n_exp_ =* 31, *n_clt_ =* 30), F = 61, M_age_ = 35	HS	4 weeks	PSS-4, SEE, System’s data	RCT	Self-regulation of addictive behaviors, adaptive coping skills, stress management
Inkster, Sarda, and Subramanian 2018 [36]	UK	Smartphone-based empathetic AI chatbot app	Mindfulness combined with behavioral techniques	*n* = 129 (high vs. low users; *n*_h_ = 108 and *n*_l_ = 21)	HS	8 weeks	PHQ-9	Quasi-experimental mixed-methods approach	Emotional resilience skills
Mehta et al., 2021 [90]	USA	AI-powered smartphone app	Breathing, gratitude, self-compassion	*n* = 4.517, F = 3687, M = 693, NB = 155, M_age_ = 28.73	HS	4 weeks	GAD-7, PHQ-9	Longitudinal observational study	Emotion regulation, cognitive control adaptive behavior
Anan et al., 2021 [107]	Japan	AI-assisted smartphone chatting app	Acceptance and mindfulness-based intervention	*n =* 94 (*n_exp_ =* 48, *n_clt_ =* 46), F = 22, Μ = 72, M_age_ = 41.8	HS	12 weeks	Linear regression analysis	RCT	Management of intense feelings
Denecke, Vaaheesan, and Arulnathan 2021 [104]	Switzerland	Mobile app with integrated chatbot	CBT and mindfulness	*n* = 21, F = 13, M = 8, M_age_ = 38.4	HS	4 weeks	UEQ	Usability testing	Emotional regulation
Sturgill et al., 2021 [105]	USA	AΙ-powered emotional intelligence and mindfulness app	Positive affirmations, gratitude exercises	*n* = 99 (*n_exp_* = 50, *n_ctl_* = 49), M_age_ = 19.9, F = 27, M = 69	HS	14 weeks, M_eng_ = 1424 min, 2 sessions per week	GAD-7, PHQ-9, TWI	RCT	Emotional awareness, emotional wellness, stress, and mood regulation
Gabrielli et al., 2021 [106]	Italy	Atena psychoeducational chatbot	Mindfulness for presence and attention	*n* = 71, F = 48, M = 23, M_age_ = 20.6	HS	Eight sessions, 4 weeks, two per week for 10 min	GAD-7, PSS-10, FFMQ	Mixed-methods proof-of-concept study	Self- and emotional awareness, negative thoughts regulation
Vertsberger, Naor, and Winsberg 2022 [126]	Israel	AI-powered conversational agent via smartphone	ACT, gratitude, breathing, self-compassion	*n =* 10.387, no gender, M_age_ = 16	HS	16 weeks, 45.39 days	WHO-5	Longitudinal study	Emotional and psychological flexibility
Naor, Frenkel, and Winsberg 2022 [140]	USA	AI-powered conversational agent via smartphone	ACT, mindfulness, and reflective exercises	*n* = 2.909, no gender, no age	HS	8 weeks–one year	WHO-5	Pragmatic retrospective analysis	Emotional well-being
Leo et al., 2022 [145]	USA	AI-based chatbot	Deep breathing, mindfulness	*n* = 61, F = 53, M = 7, NB = 1, M_age_ *=* 55	HS	8 weeks	PROMIS	Feasibility prospective cohort study	Mood and anxiety regulation
Marcuzzi et al., 2023 [121]	Norway	AI-based smartphone app	Mindfulness audio files	*n* = 294 (*n_exp_* = 99, *n_clt_* = 98, *n_clt_* = 97), M_age_ = 50.6, F = 173, M = 121	HS	12 weeks	MSK-HQ, RMQ, PSEQ, B-IPQ	RCT	Self-management of behavior
Potts et al., 2023 [139]	UK	Multilingual chatbot	Mindfulness, breathing, gratitude	*n =* 348, F = 254, M = 94, M_age_ = 30,	HS	12 weeks	SWEMWBS, WHO-5, SWLS	Pre/post-multicenter intervention study	Mental well-being

*n*: number of participants, *n*_exp_: experimental group, *n*_clt_: control group, M_age_: mean age, F: female, M: male, NB: non-binary, M_eng_: mean total engagement, PHQ-9: Patient Health Questionnaire-9, GAD-7: 7-item Generalized Anxiety Disorder, CBT: cognitive behavioral therapy, ΤWI: TestWell Wellness Inventory, ACT: Acceptance and Commitment Therapy, WHO-5: 5-item World Health Organization Well-being Index questionnaire, MBSR: Mindfulness-Based Stress Reduction, MSK-HQ: Musculoskeletal Health Questionnaire, RMQ: Roland–Morris Disability Questionnaire, PSS-4: Perceived Stress Scale, SEE: Self-efficacy for exercise scale, PSEQ: Pain Self-Efficacy Questionnaire, B-IPQ: Brief Illness Perception Questionnaire, UEQ: User Experience Questionnaire, GAD-7: 7-item Generalized Anxiety Disorder scale, PSS-10: 10-item Perceived Stress Scale, FFMQ: Five-Facet Mindfulness Questionnaire, SWEMWBS: Short Warwick–Edinburgh Mental Well-Being Scale, SWLS: Satisfaction with Life Scale, PROMIS: Patient-Reported Outcomes Measurement Information System.

**Table A2:** Summary of the studies that employed XR (VR, AR, and MR).

Study	Country	Digital Design	Mindfulness Program	Sample	Clinical Condition	Duration	Type of Measurement	Research Design	Main Improvements
Gromala et al., 2015 [143]	Canada	HMD like the Oculus Rif	MBSR	*n* = 13 (*n_exp_* = 7, *n_clt_* = 6), F = 7, M = 6, M_age_ = 49	HS	12 min	NRS	RCT	Self-regulation, resilience
Navarro-Haro et al., 2016 [114]	USA	Kaiser Electro-OpticsVR goggles	DBT mindfulness	*n* = 1, F = 1 32 years	BPD	4 sessions	DBT diary card, KIMS-Short	Case study	Self-regulation, introspection, emotional regulation
Serra Pla et al., 2017 [95]	Spain	VR goggles	MBSR	*n* = 50, *(n*_exp_ = 25, *n*_clt_ = 25)	ADHD	Four 30 min sessions	Pre-treatment, post-treatment, and at 3- and 12-months post-treatment	RCT	Self-management
Shiban et al., 2017 [112]	Germany	V6 HMD	Mindful breathing	*n* = 29, (*n*_exp_ = 15, *n*_clt_ = 14), F = 24, M = 5, M_age_ = 34.3	DEP, PB	1 session	HR, SCL, RR, ASI, FFS, FSB	RCT	Self-control
Gomez et al., 2017 [111]	Saudi Arabia	Oculus Rift DK2 VR goggles	DBT^®^ mindfulness skills training	*n* = 1, 21 years old	ANX	4 sessions	PCL-C	Case study	positive thinking, emotional regulation
Cikajlo et al., 2017 [94]	Ireland	ReCoVR System Design, 3D VR headset	MBSR	*n* = 8, M_age_ = 36	ANX	8 weeks, 30 min/per session	SWLS, MAAS	Usability study	Attention regulation and anxiety management
Seol et al., 2017 [136]	Korea	HMD, leap motion sensor, PSL-lecg2, and Falcon device	Mindfulness scenarios, breathing regulatory guidance	*n* = 5	PD	2 sessions	DASS	Usability study	Emotional stability, stress management
Roo et al., 2017 [141]	USA	MR (immersive VR, AR, and tangible interface	Mindful breathing	*n =* 12, F = 12, M_age_ = 45	HS	2 sessions	FFMQ, STAI-YA, TMS	Usability study	Increased relaxation and state mindfulness
Tarrant, Viczko, and Cope 2018 [135]	USA	Gear VR HMD powered by Samsung S7, Mindfulness in nature experience, by StoryUp VR	VR and non-VR meditations	*n* = 26 (*n_exp_* = 14, *n*_ctl_ = 12), F = 20, M = 6, M_age_ = 46.21	GAD	a brief 75 min VR meditation intervention	GAD-7, STAI EEG patterns	RCT	Self-regulation of anxiety
Navarro-Haro et al., 2019 [147]	Spain	Oculus Rift DK2 VR goggles with HMD, with head tracking	DBT mindfulness training	*n* = 39 (*n*_exp_ = 19, *n*_ctl_ = 20), F = 30, M = 9, M_age_ = 45.23	GAD	6 sessions	GAD-7, HADS, FFMQ, DERS, VAAS	RCT	Anxiety, depression, and emotion regulation
Potts et al., 2019 [142]	New Zealand	Neurofeedback AR application	Zen gardening	*n =* 12 adults	HS	Single session	EEG measurement via Muse headset	Usability study	Relaxation
Chandrasiri et al., 2019 [124]	Australia	Oculus Rift HMD	Mindfulness, decentering, curiosity	*n* = 32, *(n_exp_ =* 16, *n_clt_* = 16), M_age_ = 27.25	HS	Single session (20 min)	TMS	RCT	Receptivity to observing undesirable thoughts and feelings without interfering with them
Bossenbroek et al., 2020 [122]	Netherlands	IVR biofeedback game	Mindful breathing	*n* = 8 F = 1, M = 7, M_age_ = 14.67	ADHD	4 weeks, 6 sessions. 15 min	STAI, Likert Scale to measure disruptive classroom behavior	Single-case experimental study	Self-regulation
Chavez et al., 2020 [134]	USA	Oculus Go headset	Guided meditation	*n* = 28 (*n*_1_ = 8, *n*_2_ = 11, *n*_3_ = 10) M = 15, F = 14 M_age_ = 21.6	ADHD, DEP, ANX, BPD	1 session	STAI-6, salivary cortisol	RCT	Anxiety regulation
Mistry et al., 2020 [94]	Canada	HMD	Guided meditation	*n* = 96 (clinical sample: *n* = 26), F = 54, M = 42, M_age_ = 24.02	PTSD	1 session	LEC-5, LES, ACE, PCL-5, MEQ, TRASC, mDES, BASS,	Within-group mixed-methods study	Positive affect, emotional regulation, resilience
Yildirim and O’Grady 2020 [93]	USA	HTC Vive	Guided meditation	*n =* 45 (*n_exp_*_1_ = 15, *n_exp_*_2_ = 15, *n_clt_*_1_ = 12, *n_clt_*_2_ = 94), F = 27, M = 18 M_age =_ 20.5	HS	1 session, 10 min	SART, SMS	RCT	Self-regulation
Modrego-Alarcón et al., 2021 [108]	Spain	VR goggles	MBSR	*n* = 280 (*n*_exp_ = 93, n_clt1_, = 93, *n*_clt2_ = 94), F = 59, M = 221 M_age =_ 22.25	HS	6 weeks, once a week, 90 min per session	PSS, STAI	RCT	Emotional balance, resilience, academic engagement
Veling et al., 2021 [115]	Netherlands	Smartphone, connected to the VR HMD, VR relaxation software (https://vrelax.com/?pk_campaign=19861442586&pk_kwd=vr%20therapie&pk_source=google&pk_medium=cpc&pk_content=652068790509&gad_source=1&gclid=Cj0KCQiA4NWrBhD-ARIsAFCKwWtJKwmECx_PukSA3IQxmxb6GMUBYZP_0d_dsaUUKC_sHqj4mpTy0PMaAjxIEALw_wcB, accessed on 31 October 2023)	Guided meditation and progressive relaxation techniques	*n* = 50 (*n*_exp_ = 25, *n*_clt_ = 25), F = 33, M = 17, M_age_ = 41.6	BPD, ANX, PSY, DEP	20 (minimum of 10 min, 10 consecutive days per session)	BAI, GPTS, IDS, PSS-10, SSQ, VAS on positive and negative affective state	RCT	Positive thinking, negative thoughts management
Habak et al., 2021 [66]	Australia	MR platform VR headset	Positive visualizations	*n* = 79, F = 53, M = 23 male, NB = 3, M_age_ = 29.5	DEP	3 sessions	PANAS, BHS, SWEMWBS,	Usability study	Regulation of negative affect, positive future thinking
Viczko, Tarrant, and Jackson 2021 [116]	USA	AR phone app	Guided meditation	*n* = 41 *(n_exp_ =* 22, *n_clt_ = 19)* M_age_ = 35.4	GAD, DEP, ANX	Single session	BRUMS, EEG data collection,	RCT	Self-regulation of mood and emotion
Waller et al., 2021 [129]	Canada	VR headset powered by smartphone	Guided meditation	*n* = 82 (*n_exp_ =* 41, *n_clt_ =* 41), F = 50, M = 32, M_age_ = 22.5	HS	Single session	LES, LEC-5, ACE, PCL-5, TRASC, Mdes, BASS, MEQ, MBAS	RCT	Self-regulation of stress
Kaplan-Rakowski, Johnson, and Wojdynski 2021 [131]	USA	Oculus Go headset	Guided meditation	*n* = 61 (*n_exp_ =* 31, *n_clt_ =* 30), F = *35*, M = 26, M_age_ = 20.89	HS	Single session	Students’ test scores	RCT	Self-regulation of pre-exam anxiety
Lacey, Frampton, and Beaglehole 2022 [113]	New Zealand	Smartphone app combined with the headset that holds the smartphone and uses 360° video	Meditative techniques based on acceptance and flexibility around anxiety	*n* = 126 (*n_exp_* = 51/63 analyzed, *n_clt_*: 58/63 analyzed), M_age_ = 42.2	PB	6 weeks	SMSP, PHQ9, BFNE	RCT	Flexibility to behavioral change, optimism, self-management of negative thoughts and fears
Lunsky et al., 2022 [130]	Canada	VR meeting platform	MBSR	*n* = 37, F = 14, M = 21, NB = 2, M_age_ = 31	ASD	6 weeks, 60 min per session	DASS-21, FFMQ-SF, SCS-SF	Usability study	Stress management, connectedness
Jiang et al., 2022 [133]	USA	AR-based meditation app and biofeedback	Guided meditation	*n =* 10, M = 8, F = 2, M_age_ = 27.6	HS	5 min meditations	biosignals	Usability study	Stress management
Tarrant, Jackson, and Viczko 2022 [110]	USA	BrainLink Lite EEG headband and Oculus Go VR headset	Guided meditation	*n* = 100, *(n_exp_* = 50, *n_clt_* = 50) M_age_ = 42.1	HS	single 50 min visit	BRUMS, biofeedback	RCT	Mood regulation
Ilioudi et al., 2023 [127]	Sweden	VR HMD (an Oculus Go) running the Calm app	Βreathing, Guided relaxation	*n* = 60 (*n_e_*_xp_ = 40, *n*_clt_ = 20), F = 35, M = 25 M_age_ = 39.1	ANX, DEP, BPD	1 session	MADRS-S, BAI	Quasi RCT	Self-relaxation
Pascual et al., 2023 [132]	USA	VR-guided meditation platform, VR headset	Guided meditations using paced breathing	*N* = 32, adults	HS	4 weeks, VR users: 7 sessions, 200 sec each	PROMIS Anxiety Short Form, Heart-rate variability	RCT	Self-regulation
Cinalioglu, Sekhon, and Rej 2023 [109]	Canada	Oculus Quest 2 headset	Guided meditation	*n =* 30 (*n_e_*_xp_ = 15, *n*_clt_ = 15), aged ≥ 60	ANX	8 sessions	PSS	RCT	Self-management of depression and anxiety

*n*: number of participants, IVR: immersive virtual reality, M_age_: mean age, F: female, M: male, NB: non-binary, Exp: experimental group, HS: healthy subjects, n_Clt_: control group, n_exp_=experimental group, HMD: head-mounted display, RCT: randomized controlled trial, STAI: State-Trait Anxiety Inventory-Y, LES: life events survey, LEC-5: life events checklist for DSM-5, ACE: adverse childhood experience, PCL-5: post-traumatic stress disorder checklist for DSM-5, TRASC: trauma-related altered states of consciousness, Mdes: modified differential emotions scale, BASS: Buddhist affective states, MEQ: normative meditative experiences, BAI: Beck anxiety inventory, GPTS: green paranoid thoughts scale, IDS: inventory of depressive symptomatology, PSS: perceived stress scale, VAS: visual analog scales, SMSP: Severity Measures for Specific Phobia—Adults, BFNE: Brief Fear of Negative Evaluation, PHQ9: Patient Health Questionnaire 9, MADRS-S: Montgomery–Åsberg Depression Rating Scale-Self Assessment ΒAΙ: Beck Anxiety Inventory, DASS: Depression Anxiety Stress Scales, MBSR: Mindfulness-Based Stress Reduction, DASS-21: 21-item Depression, Anxiety & Stress Scales, FFMQ-SF: 24-item Five-Facet Mindfulness Questionnaire-Short form, SCS-SF: Self-Compassion Scale—Short Form, SWLS: Satisfaction With Life Scale, MAAS: Mindful Attention Awareness Scale, PSS: Perceived Stress Scale, DBT: Dialectical Behavior Therapy, HADS: Hospital anxiety and depression scale, FFMQ: Five facets of mindfulness questionnaire, DERS: Difficulties of emotion regulation scale, MAIA: Multidimensional assessment of interoceptive awareness BHS: Beck Hopelessness Scale, SWEMWBS: Short Warwick–Edinburgh Mental Well-Being Scale, PCL-C: PTSD CheckList-Civilian version, KIMS: Kentucky Inventory of Mindfulness Skills, STAI-6: State-Trait Anxiety Inventory-6, HR: Heart rate, SCL: skin conductance level, RR: Respiration rate, ASI: Anxiety Sensitivity Index, FFS: Fear of Flying Scale, FSB: Flying phobia screening questionnaire, TMS: Toronto Mindfulness Scale, BRUMS: Brunel Mood Scale, PSS: perceived stress score, SART: Sustained Attention to Response Task, SMS: State Mindfulness Scale, ADHD: attention-deficit/hyperactivity disorder, ASD: autism spectrum disorder, ID: intellectual disabilities, PTSD: post-traumatic stress disorder, BPD = bipolar disorder, GAD: generalized anxiety disorder, ANX = anxiety, DEP = depression, PD = panic disorder, PSY = psychosis, PB: phobia.

**Table A3:** Summary of the studies that employed BCIs and biofeedback in mindfulness training.

Study	Country	Digital Design	Mindfulness Training	Sample	Clinical Condition	Duration	Measurement	Research Design	Main Results
Kosunen et al., 2016 [100]	Finland	Neurofeedback virtual VR, Oculus Rift DK2 HMD	Focused attention, body scanning	*n* = 43, F = 26, M = 17, M_age_ = 28.7	HS	Single session	MEDEQ, ITC-SOPI	Exploratory study	Anxiety self-management
Bhayee et al., 2016 [99]	Canada	Muse^TM^ headband, mobile app	Breath-focused meditation	*n* = 26 (*n_e_*_xp_ = 13, *n*_clt_ = 13), F = 12, M = 14 M_age_ = 33	HS	6 weeks	BSI, Stroop, digit span task, FMI, PANAS, BFI WHOQOL-BREF	RCT	Attention control, inhibition, and positive mood
Cheng et al., 2016 [144]	Denmark	Smartphone app	Mindfulness meditation techniques	*n* = 10, F = 5, M = 5, 21–42 years	HS	30 min sessions	AttrakDiff	Feasibility study	Self-relaxation
Salehzadeh et al., 2017 [102]	Japan	Smartphone app	Guided meditation	*n =* 18, F = 8, M = 10, M_age_ = 27	HS	5 days	ANT, POMS, PGWBI, EEG data	Feasibility study	Attention regulation, mood regulation
Crivelli et al., 2018 [92]	Italy	Muse^TM^ headband and smartphone	Focused attention	*n* = 40, (*n_e_*_xp_ = 20, *n*_clt_ = 20), M_age_ = 23.4	ANX	4 weeks	MIDA, BRT, SRT, ERT, CRT, EEG recording	RCT	Behavioral control, attention regulation, and inhibition control
Martinez and Zhao 2018 [123]	USA	Muse^TM^ headband and iPad tablet	Focused attention	*n* = 19 (*n_e_*_xp_ = 10, *n*_Clt_ = 9), F = 11, M = 8 M_age_ = 12	HS	20 sessions, 3 min. per session	EEG assessment	RCT	Self-regulated behaviors
Antle et al., 2018 [148]	Canada	NeuroSky headset and Smartphone	Mindfulness stress reduction	*n* = 21(*n_e_*_xp_ = 9, *n*_clt_ = 12), F = 21, M_age_ = 8	HS	6 weeks, three to four times per week	Behavioral assessment	RCT	Self-regulation
Balconi, Fronda, and Crivelli 2018 [13]	Italy	Muse^TM^ headband, lowdown focus glasses, and smartphone	Focused attention	*n* = 55 (*n_e_*_xp_ = 38, *n*_clt_ = 17), M_age_ = 23.21	HS	4 weeks	STAI, BSI, BDI	RCT	Self-control, observation skills, emotional regulation
Richter et al., 2019 [103]	Canada	Muse™ headband and smartphone or tablet	Guided meditation	*n* = 55 (*n_e_*_xp_ = 27, *n*_clt_ = 28)	OCD	8 weeks	Y-BOCS, EEG assessment	RCT	Self-management of obsessions and compulsiveness
Balconi, Crivelli, and Angioletti 2019 [98]	Italy	Lowdown Focus glasses and mobile app	Breathing awareness	*n* = 50 (*n_e_*_xp_ = 38, *n*_clt_ = 12), M_age_ = 24.20	HS	3 weeks	Attentional matrices, DBQ, MFTC, Stroop test	RCT	Attention regulation
Crivelli et al., 2019 [91]	Italy	Lowdown Focus brain-sensing eyeglasses, V-Amp system, and smartphone app	Vipaśyanā meditation	*n* = 16, F = 8, M = 8, M_age_ = 44.38	HS	2 weeks, 14 sessions	EEG, STAI, PPS, POMS	Longitudinal control trial	Cognitive and affective regulation
Crivelli, Fronda, and Balconi 2019 [97]	Italy	Muse™ headband and smartphone app	Focused attention, breathing awareness	*n* = 50 (*n_e_*_xp1_ = 15, *n_e_*_xp2_ = 17, *n*_clt_ = 18), M_age_ = 22.94	HS	2 weeks	EEG, PSS-10, STAI-trait, FFMQ	Three-branch pre–post-experiment study	Attentional regulation
Millstine et al., 2019 [84]	USA	Muse™ headband and smartphone or tablet	Meditation instructions	*n* = 30 (*n_e_*_xp_ = 17, *n_e_*_xp2_ = 13), F = 30, M_age_ = 55.9	CN	2 weeks, 29 sessions	MFSI-SF, FACT-G, PSS	RCT	Self-management of emotional fatigue
Hunkin, King, and Zajac 2020 [96]	Australia	Muse^TM^ headband and iPad tablet	Breath-focused meditation	*n* = 68, F = 40, M = 28, M_age_ = 22.66	HS	2 weeks	EEG assessment, MEQ,	Crossover trial	Self-awareness, better sense of self-control
Can et al., 2020 [119]	Turkey	Unobtrusive automatic stress reduction system with consumer-grade smart bands (Empatica E4 wristband)	Guided mindfulness	*n =* 18, F = 6, M = 9, M_age_ = 28	HS	8 days	Questionnaire Self-Report Stress Data and physiological signals	Experimental study	Emotional regulation, stress management, self-recognition, acceptance, cognitive reappraisal
Schuurmans et al., 2021 [137]	Netherlands	Muse™ headband and iPad	Deep-breathing techniques	*n* = 77 (*n_e_*_xp_ = 40, *n_clt_* = 37), F = 31, M = 46, M_age_ = 15.25	PTSD	6 weeks	TRIER-C, basal ANS activity, basal HPA axis activity	RCT	Self-regulation of anxiety
Viczko, Tarrant, and Jackson 2021 [116]	USA	AR phone app	Guided meditation	*n* = 41 (*n_e_*_xp_ = 22, *n_clt_* = 19) M_age_ = 35.4	GAD, DEP, ANX	Single session	BRUMS, EEG data collection,	RCT	Self-regulation of mood and emotion
Hawley et al., 2021 [101]	Canada	Muse^TM^ headband and smartphone	Open monitoring	*n* = 71(*n_e_*_xp_ = 36, *n_clt_* = 35), M_age_ = 26	OCD, DEP, ANX	8 weeks	EEG recording, FFMQ, Y-BOCS	RCT	Less mind-wandering, reduced reactivity, self-management of obsessions
Acabchuk et al., 2021 [117]	USA	Muse^TM^ headband, Oculus Rift VR glasses, and smartphone	Self-guided meditation	*n* = 53, (*n_e_*_xp_ = 26, *n_clt_* = 27), F = 39, M = 14, M_age_ = 20.52	HS	4 weeks, 10 min daily	EEG measurements, DASS-21, MINDSENS (FFMQ)	RCT	Self-regulation
Järvelä et al., 2021 [128]	Finland	Neurofeedback VR meditation	Compassion meditation	*n* = 78, F = 45, M = 32, M_age_ = 26.08	HS	4 sessions	EEG assessment, SAM, PAI, PAU	Experimental study	Emotional awareness
Minen et al. 2021 [120]	USA	App-based HRV biofeedback (HeartMath (www.heartmath.com)	Mindful breathing	*n =* 52, (*n_exp_ = 26*, *n_clt_ =* 26), F = 46, M = 5, M_age_ = 42.2	Migraine (with a history of anxiety and depression)	8 weeks, 29 sessions	MSQv2	RCT	Mood regulation, stress management
Vekety, Logemann, and Takacs 2022 [35]	Hungary	Muse^TM^ headband and smartphone	MBSR	*n* = 31, (*n_e_*_xp_ = 15, *n_clt_* = 16), F = 16, M = 15 M_age_ = 9.92	HS	8 sessions	EEG assessment, SSRT, TMT	RCT	Impulse control
Rolbiecki et al., 2022 [118]	USA	Healium^TM^ and VR (Oculus VR headset)	Guided meditation	*n* = 15, F = 8, M = 7, Μ_age_ = 52.4	CN	12 30′ sessions	EEG assessment ESAS-r,	Feasibility study	Self-regulation of anxiety and mood
Tarrant, Jackson, and Viczko 2022 [110]	USA	BrainLink Lite EEG headband and Oculus Go VR headset	Guided meditation	*n =* 100, (*n_exp_ =* 50, *n_clt_ =* 50) M_age_ = 42.1	HS	single 50 min visit	BRUMS, biofeedback	RCT	Mood regulation
Shang et al., 2023 [138]	China	Convolutional neural networks	MBSR	*n* = 11, F = 5, M = 5, M_age_ = 35.7	HS	8 weeks	EEG assessment	Experimental study	Stress management

*n*: number of participants, M_age_: mean age, F: female, M: male, HS: healthy subjects, EEG: electroencephalography, RCT: randomized controlled trial, CN: cancer, PTSD: post-traumatic stress disorder, GAD: generalized anxiety disorder, PS: psychological disorder, OCD: obsessive-compulsive disorder, ANX: anxiety, DEP: Depression, BRUMS: Brunel Mood Scale, MEDEQ: meditation depth questionnaire, ITC-SOPI: ITC-Sense of Presence Inventory, BSI: Brief Symptom Inventory, FMI: Freiburg Mindfulness Inventory, PANAS: positive and negative affective schedule, WHOQOL-BREF: World Health Organization Quality of Life scale, BFI: Big Five Inventory, CRT: complex reaction times, STAI: State-Trait Anxiety Inventory, BSI: Brief Symptom Inventory, BDI: Beck Depression Inventory, Y-BOCS: Yale–Brown Obsessive Compulsive Scale, ANX: anxiety, DBQ: Driver Behavior Questionnaire, MFTC: Multiple Features Targets Cancellation, FFMQ: Five Facet Mindfulness Questionnaire, PSS: Perceived Stress Scale, POMS: Profile of Mood States, MFSI-SF: Multidimensional Fatigue Symptom Inventory–Short Form, FACT-G: Functional Assessment of Cancer Therapy–General, TRIER-C: Trier Social Stress Task for Children, ANS: autonomic nervous system, HΡA: hypothalamic–pituitary–adrenal axis, MEQ: Meditation Experience Questionnaire, SAM: Self-Assessment Manakins, PAI: Perceived Affective Interdependence, PAU: Perceived Affective Understanding, DASS-21: 21-item Depression, Anxiety, Stress Scale, EQ: Experiences Questionnaire, SSRT: Stop Signal Reaction Time, TMT: Trail Making Test, ESAS-r: Edmonton Symptom Assessment System revised version, ANT: Attentional Network Test, POMS: Profile of Mood State, PGWBI: Psychological General Well-being Index, SHS: Subjective Happiness Scale, MSQv2: Migraine-Specific Quality of Life Questionnaire.
